# Elucidating the role of exogenous melatonin in mitigating alkaline stress in soybeans across different growth stages: a transcriptomic and metabolomic approach

**DOI:** 10.1186/s12870-024-05101-9

**Published:** 2024-05-08

**Authors:** Yajuan Duan, Xianxu Wang, Yan Jiao, Yangyang Liu, Yue Li, Yongze Song, Lei Wang, Xiaohong Tong, Yan Jiang, Shaodong Wang, Sui Wang

**Affiliations:** 1 Key Laboratory of Soybean Biology of Chinese Education Ministry, Northeast Agricultural University, 600 Changjiang Road, Harbin, 150030 PR China; 2 School of Resources and Environment, Northeast Agricultural University, 600 Changjiang Road, Harbin, 150030 PR China

**Keywords:** Melatonin, Alkaline stress, Soybean, Transcriptomics, Metabolomics, Gene regulation

## Abstract

**Background:**

Soybean (*Glycine max*), a vital grain and oilseed crop, serves as a primary source of plant protein and oil. Soil salinization poses a significant threat to soybean planting, highlighting the urgency to improve soybean resilience and adaptability to saline stress. Melatonin, recently identified as a key plant growth regulator, plays crucial roles in plant growth, development, and responses to environmental stress. However, the potential of melatonin to mitigate alkali stress in soybeans and the underlying mechanisms remain unclear.

**Results:**

This study investigated the effects of exogenous melatonin on the soybean cultivar Zhonghuang 13 under alkaline stress. We employed physiological, biochemical, transcriptomic, and metabolomic analyses throughout both vegetative and pod-filling growth stages. Our findings demonstrate that melatonin significantly counteracts the detrimental effects of alkaline stress on soybean plants, promoting plant growth, photosynthesis, and antioxidant capacity. Transcriptomic analysis during both growth stages under alkaline stress, with and without melatonin treatment, identified 2,834 and 549 differentially expressed genes, respectively. These genes may play a vital role in regulating plant adaptation to abiotic stress. Notably, analysis of phytohormone biosynthesis pathways revealed altered expression of key genes, particularly in the *ARF* (auxin response factor), *AUX/IAA* (auxin/indole-3-acetic acid), and *GH3* (Gretchen Hagen 3) families, during the early stress response. Metabolomic analysis during the pod-filling stage identified highly expressed metabolites responding to melatonin application, such as uteolin-7-O-(2''-O-rhamnosyl)rutinoside and Hederagenin-3-O-glucuronide-28-O-glucosyl(1,2)glucoside, which helped alleviate the damage caused by alkali stress. Furthermore, we identified 183 differentially expressed transcription factors, potentially playing a critical role in regulating plant adaptation to abiotic stress. Among these, the gene *SoyZH13_04G073701* is particularly noteworthy as it regulates the key differentially expressed metabolite, the terpene metabolite Hederagenin-3-O-glucuronide-28-O-glucosyl(1,2)glucoside. WGCNA analysis identified this gene (*SoyZH13_04G073701*) as a hub gene, positively regulating the crucial differentially expressed metabolite of terpenoids, Hederagenin-3-O-glucuronide-28-O-glucosyl(1,2)glucoside. Our findings provide novel insights into how exogenous melatonin alleviates alkali stress in soybeans at different reproductive stages.

**Conclusions:**

Integrating transcriptomic and metabolomic approaches, our study elucidates the mechanisms by which exogenous melatonin ameliorates the inhibitory effects of alkaline stress on soybean growth and development. This occurs through modulation of biosynthesis pathways for key compounds, including terpenes, flavonoids, and phenolics. Our findings provide initial mechanistic insights into how melatonin mitigates alkaline stress in soybeans, offering a foundation for molecular breeding strategies to enhance salt-alkali tolerance in this crop.

**Supplementary Information:**

The online version contains supplementary material available at 10.1186/s12870-024-05101-9.

## Background

Soybean (*Glycine max*), a key economic and oilseed crop, boasts a protein content of about 40% and an oil content of around 20%. It accounts for approximately 60% of the world’s edible oil production and about 70% of plant protein consumed by humans and animals. China possesses nearly 100 million hectares of saline-alkali land, where soybean cultivation must contend with the challenging conditions of saline-alkaline stress, adverse to plant growth and development [1]. In such environments, alkali stress disrupts normal plant growth, development, and metabolic processes. High pH, osmotic stress, and disturbances in cellular pH stability, cell membrane integrity, root vigor, and photosynthetic capacity are some of the critical challenges plants face in alkaline soils, potentially leading to wilting or plant mortality [2]. The plant root system is the initial sensor of alkali stress, subsequently transmitting the stress signal to the aerial parts of the plant. Key indicators of plant seedling response to alkali stress include root length, plant height, leaf area, and photosynthetic rate [3].

To counteract alkali stress, plants enhance osmoregulation by accumulating organic compounds such as proline, and soluble sugars, thus maintaining intracellular water potential [4]. Hormonal fluctuations, involving auxin (IAA), abscisic acid (ABA), salicylic acid (SA), cytokinin (CK), and jasmonic acid (JA), are pivotal in plant adaptation to these adverse conditions [5]. In high pH environments, the accumulation and distribution of IAA and CK, essential hormones for plant growth, are particularly notable in root tips [6]. Moreover, transcription factors, especially from the AP2/ERF (APETALA2/ethylene-responsive factor), NAC (NAM,ATAF1/2,CUC1/2),WRKY, and bHLH (basic helix-loop-helix) families, play a crucial role in signal transduction under alkali stress, as evidenced by numerous transcriptome analyses indicating significant alterations in their expression levels [7–10]. While extensive research has been conducted on the molecular mechanisms of plant responses to salt stress [11–13], alkali stress has received comparatively less attention [14, 15]. Understanding plant response mechanisms to alkali stress is essential, particularly in the context of soil salinity's detrimental impact on plants.

Melatonin (N-acetyl-5-methoxytryptamine), a derivative of tryptophan, is a pinealoid substance originating in the pineal gland of animals and is also present in various plant tissues [16, 17]. Functioning primarily as a terpenoid, its synthesis predominantly occurs in chloroplasts and mitochondria, where it acts as a scavenger of reactive oxygen species [18]. Recent research has expanded our understanding of melatonin, highlighting its role in enhancing seed germination, modulating flowering time, stimulating plant growth and lateral root formation, and serving as a crucial regulator under both biotic and abiotic stress conditions [19–22]. The identification of the plant melatonin receptor CAND2/PMTR1 and the subsequent predictive modelling of its signalling pathway have led to the recognition of melatonin as a novel phytohormone [23]. In the realm of melatonin-mediated stress tolerance, studies have shown that melatonin significantly bolsters the antioxidant system and photosynthetic efficiency in soybean. It facilitates the accumulation of amino acids and their derivatives and mitigates growth, developmental, and yield losses under drought stress [24]. Maintaining ion homeostasis is a critical aspect of salt stress response, and melatonin has been found to upregulate genes like *NHX1* and *AKT1*, encoding key ion channels instrumental in this process [25]. Additionally, in the context of temperature stress, melatonin treatment has been observed to counteract the inhibitory effects on plant shoot and root growth by modulating the cellular redox state and reducing reactive oxygen species accumulation [26].

Soybean is a classic glycophyte, inherently possessing limited resilience against saline and alkaline stress [27]. Cultivating soybeans in such challenging environments markedly hampers their growth and development, often culminating in significant yield reductions [28]. While previous research has underscored melatonin's pivotal roles in plant growth [29, 30], seed germination [31], and adaptation to environmental stressors [32], its specific effects and underlying mechanisms in mitigating alkali stress in soybeans remain inadequately understood. This study aims to decipher the regulatory network of exogenous melatonin in counteracting alkali stress in soybeans. We focused on the vegetative growth and pod-filling stages of the Zhonghuang 13 soybean variety, subjected to alkali stress and treated with exogenous melatonin. A comprehensive array of analytical tools, including transcriptomic and metabolomic analyses, alongside physiological and biochemical indices, were employed. This comprehensive approach not only elucidates the role of melatonin in soybean stress response but also sets a foundational framework for the molecular breeding of salinity-tolerant soybean varieties.

## Methods

### Plant materials and processing conditions

The subject of this study was the soybean cultivar 'Zhonghuang 13'(presented by the Institute of Crop Science, Chinese Academy of Agricultural Sciences), characterized by a fertility period of approximately 130 to 135 days. Melatonin, obtained from Shanghai Yuanye Biotechnology Company (Songjiang District, Shanghai, China), was used with a purity exceeding 99% (B21269-20 mg). The experiment was structured into four distinct treatment groups: control with normal watering (CK, water 200 ml), alkali stress (AS, pouring 200 ml of 50mmolꞏL^−1^ NaHCO_3_ solution), alkali stress combined with melatonin (AS + MT, 200 ml of 50 mmol·L^−1^ NaHCO_3_ mixed with 100 μmolL^−1^ melatonin was poured), and melatonin alone (MT, 200 ml of 100 μmolL^−1^ melatonin solution was poured in). Plants were grown in pots in a greenhouse at Northeast Agricultural University (temperature at vegetative growth stage 16 °C-27°C, humidity 45%-56%, temperature at pod-filling stage 21 °C-31°C, humidity 42%-51%, average sunshine duration 8 h). Soybean plants were treated once a day starting from the V3 stage at the vegetative growth stage and the R5 stage at the pod-filling stage by spraying the foliage of plants containing melatonin treatments (AS, AS + MT) with the same concentration of melatonin at the same time as the solution was applied to the soil until the droplets dripped from the leaves. Following the initial melatonin application in both vegetative and pod-filling stages, parallel control samplings were conducted at predetermined intervals under each treatment condition, specific sampling time points are shown in the supporting information in the appendix. At the vegetative growth stage, fully expanded soybean leaves were taken from each treatment to be used as samples for various indexes and transcriptome sequencing. At the pod-filling stage, full and uniform pods were selected as transcriptome sequencing samples for each treatment, and the grains were used for physiological indexes and leaves for photosynthetic indexes. All samples were immediately flash-frozen in liquid nitrogen and preserved at -80 °C. To ensure statistical robustness, each treatment group consisted of at least three biological replicates.

### Measurement of physiological indicators

Plant height and root length were measured using the straightedge method, while leaf area was measured using the square methods [33]. Superoxide dismutase activity (SOD) was estimated using the superoxide dismutase typed assay kit (A001-2–2), malondialdehyde content (MDA) using the plant malondialdehyd assay kit (A003-3–1), and soluble sugar level using the plant soluble sugar content test kit (A145-1–1) supplied by the Nanjing Jiancheng Bioengineering Institute (Xuanwu district, Nanjing, China). All samples used for the assays were cryogenically frozen in liquid nitrogen.

These biochemical parameters were meticulously measured using a microplate reader. All photosynthetic parameters were measured using a portable photosynthesis meter (CID Bio-Science,Inc.Cl-340). To improve the reliability of our findings, three biological replicates were performed for each treatment and three technical replicates for each sample.

### Transcriptomic sequencing and differential gene expression analysis

The collected samples were preserved on dry ice and handed over to Frasergen Bioinformatics Co., Ltd. (Donghu Hi-Tech Development Zone, Wuhan, China) to complete the RNA sequencing process, detailed process is provided in the appendix [34–36]. Upon receiving the high-throughput sequencing raw data, quality assessment was conducted using FastQC (v0.11.7). Subsequently, high-quality clean reads were isolated for assembly and analysis using Fastp (v0.23.1). These clean reads were then aligned to the Zhonghuang 13 reference genome using Kallisto (v0.46.2) software, facilitating the quantification of transcripts per million (TPM) to determine gene expression levels. For the identification of differentially expressed genes (DEGs), a false discovery rate (FDR) control was employed to set the *P*-value threshold. An absolute value of log_2_ fold change ≥ 1 and an FDR significance score of less than 0.05 were the criteria for DEG selection. Further, gene ontology (GO) and kyoto encyclopedia of genes and genomes (KEGG) enrichment analyses were executed using OmicShare tools (www.omicshare.com/tools). The genetic trends during the pod-filling stage were analyzed using STEM software.

### Metabolomic analysis by ultra-high performance liquid chromatography-tandem mass spectrometry (UPLC-MS/MS)

Sample collection and processing: metabolomic samples were collected on the tenth day post-treatment during the pod-filling stage of the soybean. On the tenth day after treatment, metabolome samples were collected when the grains were at the pod-filling stage of soybean under the four experimental conditions (control, alkali stress, alkali stress with melatonin, and melatonin alone). A total of 12 samples, comprising three biological replicates for each treatment, were acquired and sent to Wuhan Maiteville Biotechnology Co., Ltd (Hongshan District, Wuhan, China), detailed process is provided in the appendix. for metabolomic analysis, detailed process is provided in the appendix [37, 38]. The metabolomic dataset was subjected to rigorous statistical analysis, including correlation analysis and principal component analysis (PCA) to discern patterns and relationships among the different treatment groups. Orthogonal partial least squares discriminant analysis (OPLS-DA) was utilized to identify metabolites that exhibited significant variation between the treatments. Metabolites were selected based on a fold change criterion, specifically those with a Fold Change ≥ 2 or ≤ 0.5. Subsequent KEGG pathway enrichment analysis was conducted on these differentially abundant metabolites (DAMs). Additionally, pearson correlation coefficients were calculated between DEGs and DAMs using R language. This was followed by the creation of advanced correlation network maps using Cytoscape software, providing a holistic view of the interplay between gene expression and metabolic changes.

### Weighted gene co-expression network analysis (WGCNA)

For the construction of weighted gene co-expression networks, we utilized the WGCNA package in R software. This analysis began with the formation of expression profile matrices based on the gene expression data from all collected samples. In an effort to focus on biologically significant genes, those with the highest expression TPM less than 1 were excluded, under the premise that genes with uniformly low expression across all treatments may not hold substantial biological relevance. The co-expression networks within key modules were subsequently visualized and analyzed using Cytoscape software. This approach allowed for the identification of potential gene clusters and networks that are critically involved in the soybean's response to alkali stress and melatonin treatment.

### Validation of RNA-seq by quantitative real-time PCR (RT-qPCR)

For the validation of RNA sequencing results, total RNA from leaves or pods was extracted using the universal total plant RNA rapid extraction kit (Bio Teke, RP3302, Wuxi, China). The purified RNA was then reverse transcribed into cDNA using primeScript™ RT reagent kit with gDNA eraser (RR047A), serving as the template for subsequent RT-qPCR analysis. Quantitative PCR was performed utilizing the SYBR Green method (TOYOBO, SYBR Green Realtime PCR Master Mix, QPK-201, QPK-201 T). The specifics of the primers used are detailed in Table S1. The relative transcript levels of the genes of interest were quantified using the 2^−ΔΔCt^ method. Each sample underwent analysis with three independent biological replicates to ensure statistical robustness. To address potential variability arising from the use of a single housekeeping gene, this study employed three housekeeping genes. The relative expression changes of the target genes were evaluated based on the geometric mean of the Ct values of these three housekeeping genes, thereby providing a more stable and reliable internal reference for gene expression normalization.

### Statistical analysis

The statistical analysis was conducted using one-way analysis of variance (ANOVA; *P* < 0.05; Duncan test) in SPSS 22.0 software. All data were presented as the mean value ± standard deviation (SD).

## Results

### Impact of exogenous melatonin on soybean morphology and physiology under alkaline stress

This study evaluated the influence of exogenous melatonin on soybean’s morphological, photosynthetic, and antioxidant responses under four distinct treatments. Morphological indicators revealed notable differences: at the vegetative growth stage, soybean plant height and leaf area decreased by 12.8% and 16.4%, respectively, under alkali stress (AS) compared to the control (CK). However, the AS combined with melatonin (AS + MT) treatment resulted in a smaller reduction in leaf area by 7.4%, with no significant difference in plant height relative to CK (Table S2).

**Fig. 1:**
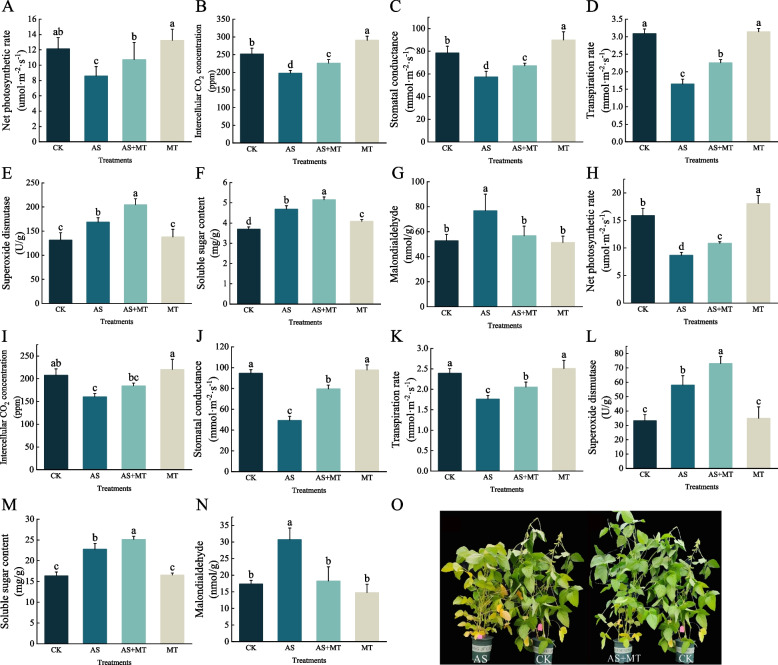
Assessment of physiological parameters and phenotypic morphology in soybean under varied treatments during the vegetative growth and pod-filling stages. **A**, **H** Net photosynthetic rate. **B**, **I** Intercellular CO_2_ concentration. **C**, **J** Stomatal conductance. **D**, **K** Transpiration rate. **E**, **L** Activity of superoxide dismutase (SOD). **F**,** M** Soluble sugar content. **G**, **N** Malondialdehyde (MDA) levels. **O** Phenotypic morphology of soybean plants at the pod-filling stage. Note: Illustrated with error bars representing standard deviation. The difference significance was examined using the Duncan’s test. Different letters denote statistically significant differences between treatments at the 0.05 level

The results of photosynthesis and physiological indexes of soybean leaves at vegetative growth stage under each treatment are shown as follows, photosynthesis and physiological indices were significantly improved under AS + MT treatment compared to AS treatment, and significant differences were observed among all indices. Compared to AS treatment, the AS + MT treatment exhibited a 25.0% increase in net photosynthetic rate and a 14.3% rise in intercellular CO_2_ concentration (Fig. 1A, B); stomatal conductance and transpiration rate were enhanced by 17.2% and 36.8%, respectively (Fig. 1C, D); SOD activity and soluble sugar content increased by 21.3% and 9.8% (Fig. 1E, F); MDA content decreased by 26.2% (Fig. 1G).

In the determination of photosynthetic indexes of leaves under various treatments at the pod-filling stage of soybean, these enhancements were more pronounced: in the AS + MT treatment compared to the AS treatment, net photosynthetic rate and intercellular CO_2_ concentration were increased by 25.2% and 15.0%, respectively (Fig. 1H, I); stomatal conductance and transpiration rate saw substantial increases of 61.8% and 16.9% (Fig. 1J, K); in the determination of physiological indices of grains under each treatment, SOD activity and soluble sugar content were elevated by 25.8% and 10.3%, respectively (Fig. 1L, M); and MDA content reduced by 46.5% (Fig. 1N), Most of all indicators reached the level of significant difference. Ten days following the initiation of alkali stress and melatonin treatment, significant morphological differences were observed during the pod-filling stage. AS-treated plants exhibited a higher degree of leaf yellowing compared to CK, whereas the AS + MT treatment showed no significant difference in leaf yellowing (Fig. 1O). Furthermore, the application of melatonin in conjunction with alkali stress, as well as melatonin treatment alone, significantly improved the soybean yield per plant (Fig. S1).

### Overview of transcriptome data

To elucidate the impact of exogenous melatonin on soybean during various growth stages under alkali stress, a detailed transcriptome analysis was conducted. A total of 27 libraries were constructed from nine soybean varieties at the vegetative growth stage, each with three biological replicates, resulting in 183.14 Gb of raw sequencing data. Post-quality filtering, 152.28 Gb of high-quality clean data was obtained. The sequencing quality was robust, evidenced by Q20 and Q30 values of each library being greater than or equal to 97.17% and 90.65%, respectively, and a GC content in the range of 42.14% to 46.12% (Table S3). During the soybean pod-filling stage, a total of 273.09 Gb of raw data was acquired from 17 samples across four treatments at five different time points. Each sample had three biological replicates, with an additional five replicates for the control group (CK). After quality filtration, 234.37 Gb of clean data was secured. The integrity of this data set was further confirmed with Q20 and Q30 values of each library exceeding 97.70% and 90.89%, respectively, and GC content varying between 43.31% and 47.30% (Table S4). To enhance analysis accuracy, a within-group sample correlation criterion of ≥ 0.8 was applied. This process ensured that at least two replicates were retained for each sample. These meticulous quality control measures affirm that the sequencing data attained are of sufficient caliber for subsequent in-depth analysis.

### Differential gene expression analysis

**Fig. 2:**
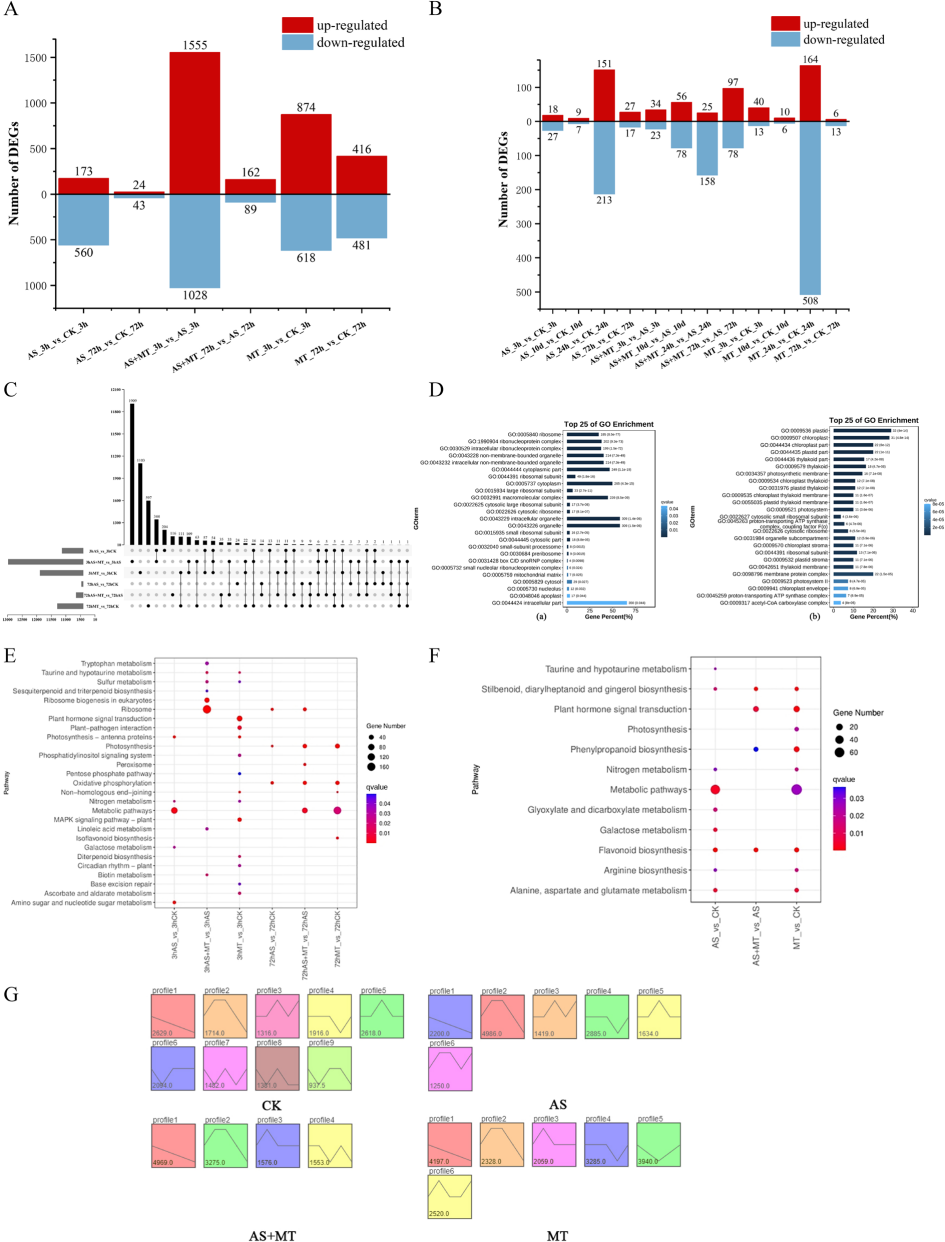
Differential gene expression and pathway enrichment analysis in soybean under various treatment combinations during vegetative growth and pod-filling stages. **A** DEGs profile at the vegetative growth stage: This panel illustrates DEGs identified in all comparative analyses at the vegetative growth stage of soybean. **B** DEGs profile at the pod-filling stage: depicting DEGs from all comparison combinations during the soybean’s pod-filling stage. **C** UpSet plot of temporal DEGs: displaying the intersection of DEGs at different time points between the AS + MT vs AS groups. **D** GO enrichment analysis: panel (a) presents GO terms enriched in the AS + MT_3h vs AS_3h comparison, while panel (b) illustrates enriched GO terms in the AS + MT_72h vs AS_72h comparison. **E** KEGG pathway enrichment during vegetative growth: showcasing significantly enriched KEGG signaling pathways in each comparison combination. The horizontal axis represents different comparison groups, and the vertical axis indicates the enriched KEGG pathways. Circle size correlates with the number of genes enriched in each pathway, while color denotes the significance level of enrichment, with blue indicating lower and red indicating higher significance. **F** KEGG pathway enrichment during pod-filling stage: highlighting significantly enriched KEGG signaling pathways in the comparison combinations at this stage. **G** Trend analysis during pod-filling stage: detailing the expression trend analysis of soybeans based on the short time-series expression miner (STEM) method

In our comprehensive transcriptome analysis of 24 soybean samples at the vegetative growth stage, we identified a total of 6,023 DEGs in response to exogenous melatonin under alkali stress. Notably, the comparison between the AS + MT_3h and AS_3h groups revealed the highest number of both up-regulated and down-regulated DEGs (Fig. 2A). This finding suggests that exogenous melatonin swiftly modulates plant responses to alkali stress within a short time frame. Moreover, employing the venn diagram function within TBtools, we identified 33 common DEGs between the AS + MT treatment vs AS treatment groups across two distinct time points (Fig. 2C). These genes are likely continuously involved in the alleviation of alkali stress in soybeans during the vegetative growth stage through the action of exogenous melatonin. At the pod-filling stage, we utilized a similar method for DEG screening, 12 sets of DEG comparison combinations were derived. A total of 1,778 DEGs were discerned across all samples, encompassing 637 up-regulated and 1,141 down-regulated genes (Fig. 2B). The temporal analysis of DEGs in both vegetative growth and pod-filling stages illustrated a pattern of an initial increase followed by a rapid decrease in DEG numbers over time. This pattern potentially reflects the dynamic process of plant adaptation to abiotic stress (Fig. S2).

### Functional annotation and enrichment analysis of DEGs

DEGs identified from comparison combinations during the vegetative growth stage were subjected to GO functional enrichment analysis. This analysis revealed 145 and 236 GO terms reaching significant levels (qvalue < 0.05) under AS vs AS + MT, respectively. Key functional categories include oxidoreductase activity (GO:0016491), redox processes (GO:0055114), superoxide dismutase activity (GO:0004784), and cellular response to osmotic stress (GO:0071470), as shown in Fig. 2D. These results suggest enhanced adaptability of melatonin-treated plants to abiotic stresses, potentially through modulated metabolic processes and hormonal responses. A total of 6,023 DEGs from the vegetative growth stage were mapped onto 440 KEGG pathways. Notably, 13 pathways were significantly enriched (qvalue < 0.05) in the AS + MT vs AS comparison, including ribosome biogenesis in eukaryotes (ko03008), photosynthesis (ko00195), peroxisome (ko04146), and metabolic pathways (ko01100), as depicted in Fig. 2E.

The response of soybeans to exogenous melatonin during the pod-filling stage under alkali stress diverged significantly from that observed at the vegetative growth stage. Due to a reduced number of DEGs during the pod-filling stage, gene expression trends across five time points were analyzed using the short time-series expression miner (STEM) method (Fig. 2G). Six expression profiles were identified as significant in the AS treatment trend analysis. GO enrichment for profile 3 highlighted terms related to plant adversity and flavonoid biosynthesis, such as responses to abiotic stimuli and redox processes. In the AS + MT treatment trend analysis, GO terms associated with plant salt tolerance, like cellular hypertonic salinity response and sterol metabolic processes, were enriched in profile 2. These trends align with the early perception and signaling of adversity stress in plants. Notably, the DEGs in the AS + MT vs AS comparison were significantly enriched in three KEGG pathways: flavonoid biosynthesis (ko00941), phytohormone signaling (ko04075), and phenylpropanoid biosynthesis (ko00940), as illustrated in Fig. 2F.

### Melatonin’s modulation of phytohormone synthesis and signaling in soybean

**Fig. 3:**
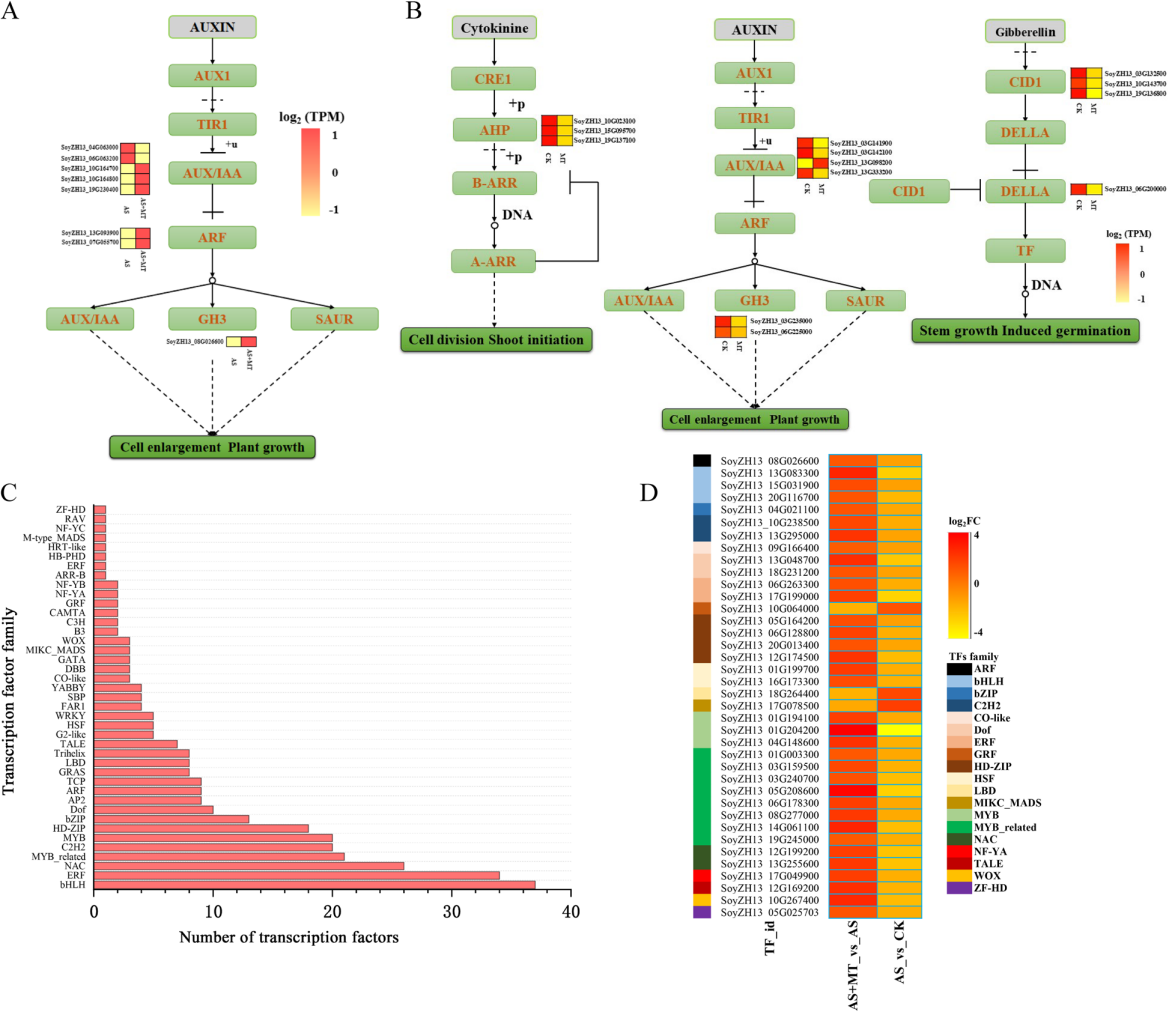
DEGs in soybean’s growth hormone signaling pathways modulated by melatonin. **A** Melatonin-induced DEGs in growth hormone signaling during vegetative growth stage: this panel illustrates the differential gene expression induced by melatonin in the soybean vegetative growth stage’s growth hormone signaling pathway. **B** Melatonin-induced DEGs in growth hormone signaling during pod-filling stage: showcasing the DEGs influenced by melatonin in the growth hormone signaling pathway of soybean during the pod-filling stage. **C** TFs encoded by DEGs: depicting 319 differentially expressed genes that encode TFs. **D** TF expression modulation by melatonin: presenting the expression levels of 38 TFs that are up- or down-regulated under alkaline stress conditions in response to melatonin treatment

Phytohormones are pivotal in plant growth, development, and stress resilience. To investigate melatonin’s role in modulating other phytohormones during soybean’s resistance to alkali stress, we analyzed gene expression in phytohormone pathways at both the vegetative growth and pod-filling stages. Our analysis revealed that 185 DEGs were involved in the phytohormone pathways during the vegetative growth stage. These DEGs are implicated in the signal transduction of plant hormones such as IAA, ABA, and JA. In the AS + MT treatment, alterations in the auxin signal transduction pathway were observed, with six genes up-regulated and two down-regulated. Notably, the up-regulated genes *SoyZH13_08G026600* and *SoyZH13_13G093900* are part of the *auxin response factor* (ARF) gene family. Additionally, the genes *SoyZH13_10G164700*, *SoyZH13_10G164800*, and *SoyZH13_19G230400*, belonging to the auxin/indole-3-acetic acid (*AUX*/*IAA*) gene family, and the *SoyZH13_07G055700* gene from the *GH3* gene family, were also identified (Fig. 3A). In the phytohormone signaling pathways of melatonin-treated soybeans during the pod-filling stage, a predominant down-regulation of genes in the IAA, cytokinin (CTK), and gibberellin (GA) signal transduction pathways was observed (Fig. 3B). The down-regulated genes primarily belong to the *AUX*/*IAA* and *GH3* families involved in growth hormone signaling, the *AHP* family in cytokinins, and the *GID1* and *DELLA* families in gibberellins.

### Melatonin-induced modulation of transcription factors during soybean’s vegetative growth stage

Given the extensive array of DEGs observed at the vegetative growth stage, our focus shifted towards identifying key transcription factors (TFs) that mediate melatonin's alleviating effects on soybean under alkali stress. Our analysis identified a total of 319 DEGs encoding transcription factors (Fig. 3C). The categorization of these DEGs revealed that in the AS + MT vs AS comparison, there were 138 up-regulated and 45 down-regulated TFs. These belonged to several transcription factor families, including AP2/ERF, bHLH, MYB, WRKY, NAC, and C2H2. Furthermore, 35 transcription factors were found to be down-regulated under AS treatment compared to CK but were up-regulated upon melatonin treatment in AS + MT. Additionally, three transcription factors, initially down-regulated between AS and CK treatments, were up-regulated by melatonin in the AS + MT treatment. These transcription factors span across families such as ERF, bHLH, MYB, NAC, MYB-related, and C2H2 (Fig. 3D).

### Metabolomic variations in soybean grains during the pod-filling stage under alkali stress and melatonin treatment

**Fig. 4:**
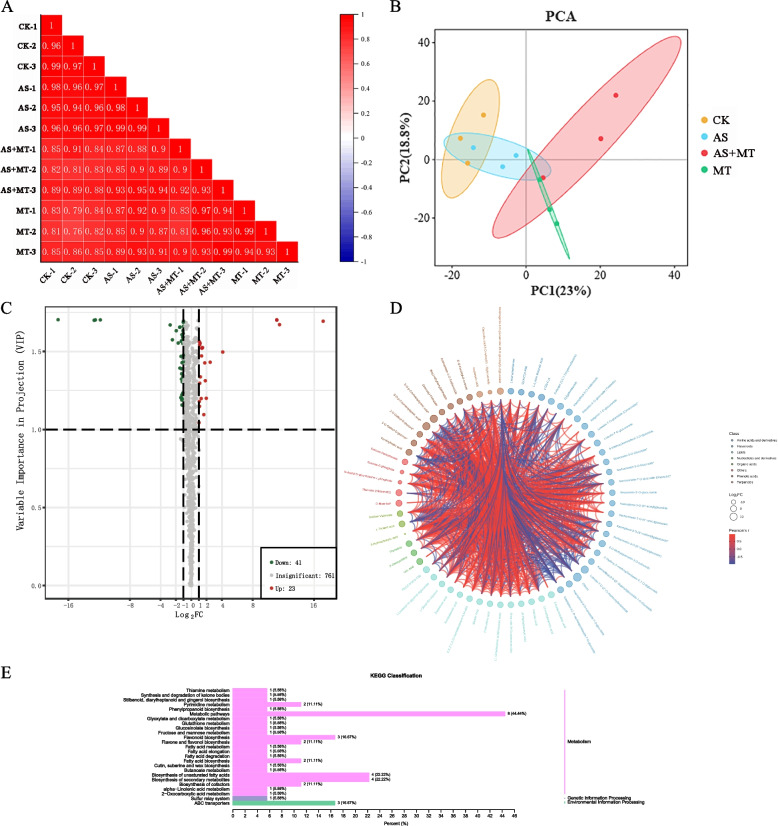
Comprehensive analysis of metabolomic alterations in soybean under alkali stress and melatonin treatment. **A** Sample correlation analysis: illustrating the correlation between replicates across different treatments, affirming the consistency and repeatability of the metabolomic data. **B** Principal component analysis (PCA): this plot delineates the distinct clustering of samples based on treatment groups, highlighting the metabolic diversity induced by different treatments. If the significance value *P* ≤ 0.05, the correlation coefficient matrix is considered not to be a unitary matrix and can be subjected to principal component analysis. **C** differentially expressed metabolite volcano plots: displaying the distribution and significance of DAMs between the AS and AS + MT groups. **D** Chord diagram of differentially accumulated metabolites: visualizing the relationships and abundance of all DAMs identified in AS and AS + MT treatments. **E** KEGG pathway classification: mapping the DAMs to specific KEGG pathways, elucidating the metabolic pathways influenced by AS and AS + MT treatments

Metabolite detection in repeat samples across each treatment group exhibited a highly significant correlation, affirming the repeatability and reliability of our sample data (Fig. 4A). Principal component analysis further delineated clear separation among the four treatment groups, validating the dataset's suitability for in-depth analysis (Fig. 4B). Across the treatment groups, we identified 825 metabolites, with 64 being DAMs between the AS treatment and AS + MT. This subset includes 23 up-regulated and 41 down-regulated metabolites (Fig. 4C) (Table S5). Correlation analysis of DAMs revealed Luteolin-7-O-(2''-O-rhamnosyl)rutinoside and Hederagenin-3-O-glucuronide-28-O-glucosyl(1,2)glucoside as highly correlated metabolites in both AS and AS + MT treatments. These compounds, belonging to flavonoids and terpenoids respectively, are key secondary metabolites in plants (Fig. 4D). Co-localization analyses of DEGs and DAMs revealed significant enrichment in the flavonoid biosynthesis (ko00941), phenylpropanoid biosynthesis (ko00940), and biosynthesis of secondary metabolites (ko01110) KEGG pathways in both AS and AS + MT treatments (Fig. 4E). To elucidate the impact of alkaline stress on the hormonal dynamics of soybean during the pod-filling stage and to explore the role of melatonin, we measured the hormone content in soybeans subjected to various treatments. A total of 39 plant hormone metabolites were quantified across the samples. In the comparative analysis between the AS treatment and the AS + MT, five DAMs were identified. These included Indole-3-acetyl glutamic acid (Auxin), Dihydrozeatin-O-glucoside riboside (Cytokinin, CK), ortho-Topolin (CK), Gibberellin A53 (GA), and 5-Deoxystrigol (Strigolactone, SL). Relative to the AS treatment, the AS + MT treatment exhibited increased levels of ortho-Topolin and Gibberellin A53, while showing decreased concentrations of Indole-3-acetyl glutamic acid, Dihydrozeatin-O-glucoside riboside, and 5-Deoxystrigol. These findings suggest that melatonin treatment under alkali stress conditions modulates the hormonal balance in soybean, potentially contributing to enhanced stress tolerance (Table S6).

### Network analysis elucidates DEGs linked to melatonin’s alkali stress response in soybean across fertility stages

**Fig. 5:**
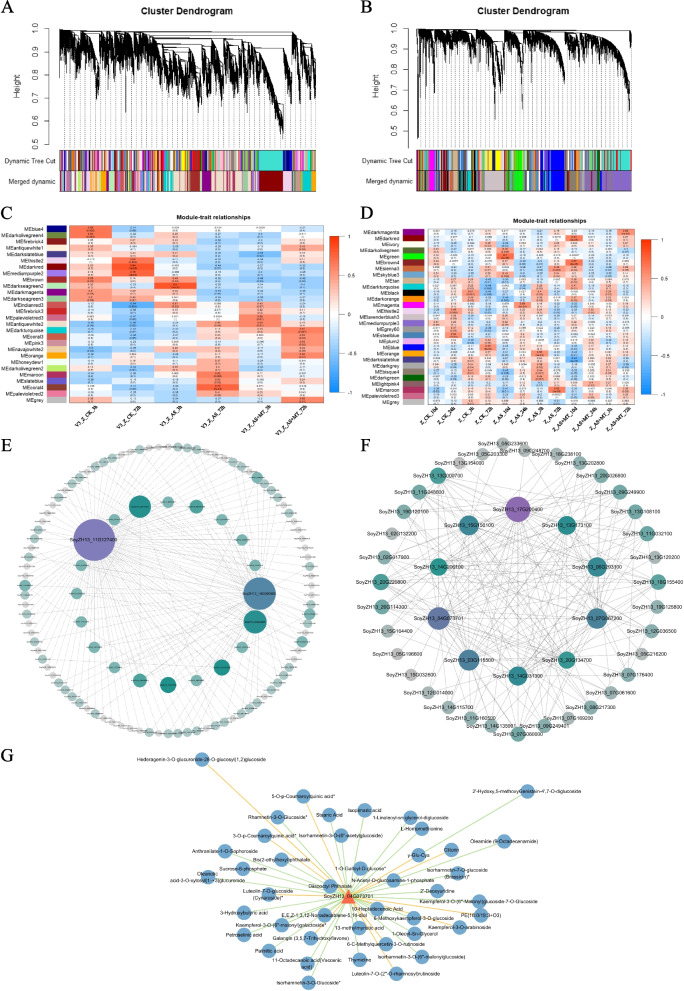
WGCNA highlighting the impact of melatonin on soybean’s response to alkali stress. **A** Hierarchical clustering tree and expression modules at the vegetative growth stage: this panel depicts the clustering of gene expression modules identified during the vegetative growth stage. **B** Hierarchical clustering tree and expression modules at the pod-filling stage: illustrating the clustering of gene expression modules observed during the pod-filling stage. **C** Gene co-expression network in the vegetative growth stage: showcasing the correlation between different treatments and the identified modules at this stage. **D** Gene co-expression network in the pod-filling stage: demonstrating the correlation between various treatments and the identified modules during the pod-filling stage. **E** Hub gene networks in the darkturquoise module: depicting the gene network and connectivity of key hub genes within the darkturquoise module identified at the vegetative growth stage. **F** Hub gene networks in the brown4 module: illustrating the gene network and connectivity of central hub genes within the brown4 module observed at the pod-filling stage. Note: The size of each node in the network represents the gene connectivity, while different colors indicate the weight value of each connection. **G** Gene-metabolite correlation network: depicting the network of interactions between the hub gene *SoyZH13_04G073701* and various metabolites. Orange triangles represent the gene, blue circles denote metabolites, with orange lines indicating positive correlations and green lines signifying negative correlations

To decipher the gene regulatory networks underpinning soybean's response to alkali stress in the presence of melatonin (AS + MT), we conducted a WGCNA using transcripts per million (TPM) values from various treatments. This analysis identified 28 distinct modules at the vegetative growth stage and 29 modules at the pod-filling stage, based on similar expression patterns (Fig. 5A and B). Notably, the darkturquoise and brown4 modules demonstrated a high correlation with the AS + MT treatment across both stages (Fig. 5C and D). Within these modules, the hub gene *SoyZH13_11G127400* was identified as a crucial regulator of melatonin’s response to alkali stress during the vegetative growth stage. This gene encodes bHLH transcription factors, pivotal in processes such as cell growth, proliferation, metabolism, and circadian rhythms (Fig. 5E). Bioinformatics and sequence homology analyses revealed that the pod-filling stage hub gene *SoyZH13_17G200400* encodes the E3 ubiquitin-protein ligase KEG. KEG plays multifaceted roles in plant development and is involved in both ABA and JA signaling pathways. Another significant hub gene at the pod-filling stage, *SoyZH13_04G073701*, is implicated in regulating DNA repair ATPase-related family proteins (Fig. 5F). The correlation network plot emphasized that the hub gene *SoyZH13_04G073701*, identified at the pod-filling stage, positively regulates the terpene metabolite Hederagenin-3-O-glucuronide-28-O-glucosyl(1,2)glucoside, and negatively regulates the flavonoid metabolite 2'-Hydoxy,5-methoxyGenistein-4',7-O-diglucoside (Fig. 5G).

### qRT-PCR validation of RNA-Seq findings in soybean under alkali stress and melatonin treatment

To corroborate the RNA-seq data, eight DEGs and three housekeeping genes were selected for qRT-PCR validation at both the vegetative growth and pod-filling stages. The qRT-PCR results demonstrated expression trends congruent with those observed in the RNA-Seq analysis (Fig. S3). This concordance substantiates the reliability and accuracy of our RNA-Seq findings, confirming the identified transcriptional changes in soybean under alkali stress and melatonin treatment.

## Discussion

Salt-alkali stress is a critical environmental factor that impairs plant growth, development, and geographical distribution, significantly affecting crop yield and quality. This stress severely disrupts normal plant growth and biochemical metabolism, with notable impacts on root surface area, leaf area, and photosynthetic rate—key parameters of plant seedling biomass response to salt-alkali stress [39]. The plant photosynthetic system is particularly vulnerable to saline stress. Excessive accumulation of Na^+^ interferes with CO_2_ diffusion through stomata and chloroplasts, thereby inhibiting photosynthesis [40]. Additionally, alkali stress impairs plant growth by diminishing photosynthetic pigments and damaging photosynthetic structures. This reduction in photosynthetic area per plant, due to inhibited chlorogenesis under saline and alkaline conditions, leads to decreased carbon assimilation [41]. Our study corroborates these findings, as soybean plants under alkali stress exhibited reduced height and leaf area during the vegetative growth stage, and severe yellowing and senescence during the pod-filling stage (Table S2, Fig. 1O).

Melatonin, recognized as a novel phytohormone, is abundantly present in plant tissues and exhibits a wide range of physiological functions. It enhances seed germination, regulates plant growth and development, and influences senescence and mortality [42, 43]. As a potent antioxidant, melatonin can ameliorate oxidative damage in plants, thereby improving plant resilience to adverse conditions [44]. Our findings indicate that melatonin application, particularly at the pod-filling stage, effectively increased soybean yield per plant (Fig. S1). This yield enhancement is attributable to melatonin’s ability to mitigate growth inhibition [45]. We observed that melatonin treatment resulted in lower levels of cytokinin-like and gibberellin-like metabolites compared to the control, as evidenced by hormone metabolite assays (Table S6). Cytokinins play a role in inhibiting chlorophyll degradation and delaying leaf senescence [46], while gibberellins are crucial in regulating processes like seed germination and fruit ripening [47].

The application of exogenous melatonin under alkali stress was found to influence the expression of various genes and the accumulation of related metabolites. According to our transcriptome data analysis, melatonin induces the up-regulation of genes associated with superoxide dismutase activity and redox processes (Fig. 2D). Upon sensing abiotic stress, melatonin can rapidly enhance the activities of various antioxidant enzymes and stress tolerance-related genes, activating downstream signaling pathways [48]. A previous study on cassava demonstrated that the interaction between two enzymes of the melatonin synthesis pathway (MeTDC2 and MeASMT2) and ascorbate peroxidase (MeAPX2) plays a crucial role in regulating redox homeostasis and tolerance to alkali stress [49]. In line with these findings, our RNA-seq data analysis identified differential expression of eight genes in the growth hormone signaling pathway in the early stage of alkali stress under the AS + MT treatment (Fig. 3A). These genes are from the *ARF*, *AUX*/*IAA*, and *GH3* gene families, which are integral to growth hormone response and regulation. The *ARF* family, serving as early response genes to growth hormones, senses upstream hormonal signals and regulates the transcription of downstream hormone-inducible genes, thereby controlling plant growth and development [50]. Studies on chickpea under abiotic stress conditions have shown significant up-regulation of certain *ARF* genes under salt, drought, and cold stress, underscoring their importance in plant stress resistance [51]. The Aux/IAA proteins, known to form heterodimers with ARF, modulate the transcriptional regulatory function of ARF [52], while GH3 family proteins maintain intracellular hormone levels through feedback regulation. GH3 is also implicated in regulating the JA and light signaling pathways during photomorphogenesis in rice [53], as well as influencing hormone content [54].

Understanding the metabolic pathways and regulatory factors through which melatonin alleviates alkali stress is crucial for enhancing stress tolerance in soybeans. Our study, utilizing WGCNA of differential genes at the pod-filling stage, identified highly connected hub genes. A significant finding in our study is the identification of the hub gene *SoyZH13_17G200400*, which encodes the E3 ubiquitin-protein ligase KEG. This discovery aligns with prior research in *A. thaliana*, which has demonstrated the critical role of EDR1 (Enhanced Disease Resistance 1) as a key negative regulator of plant immunity. Notably, the recessive missense mutant *keg-4*, uncovered through a forward genetic screen, was found to suppress the *edr1*-mediated resistance. In the *edr1* mutant background, ubiquitination by KEG results in increased levels of the MKK4 and MKK5 proteins, thereby amplifying the plant's immune response [55]. Mitogen-activated protein kinase (MAPK) pathways, highly conserved signaling modules, are integral in regulating plant immune responses [7]. Plants respond to alkali stress by modulating MAPK and Ca^2+^-dependent protein kinase (CDPK) osmotic stress signaling pathways [56]. Another critical hub gene, *SoyZH13_04G073701*, influences DNA repair ATPase-related family proteins and may be associated with seed protein and oil content [57]. Joint transcriptomic and metabolomic analyses demonstrated that *SoyZH13_04G073701* positively regulates the metabolite Hederagenin-3-O-glucuronide-28-O-glucosyl(1,2)glucoside, a triterpene saponin. Triterpenes are ubiquitous hydrocarbons in plants, serving as vital secondary metabolites in plant growth and development [58]. Oleoresin endolipids, derived from triterpenes, regulate plant growth, and sterols, also triterpene derivatives, are essential components of cell membranes [59]. In summary, *SoyZH13_04G073701* likely plays a pivotal role in the melatonin-mediated mitigation of alkali stress at the pod-filling stage in soybean. This effect is potentially achieved through direct regulation of Hederagenin-3-O-glucuronide-28-O-glucosyl(1,2)glucoside synthesis, underscoring the multifaceted impact of melatonin on plant stress responses.

Our transcriptomic analyses revealed a notable disparity in the number of DEGs between the vegetative growth and pod-filling stages. This variation may be attributed to several factors. Firstly, the DEGs from these two stages originate from distinct tissue types—leaves and seeds—which have differing physiological and metabolic profiles. Each cell type exhibits unique responses to alkali stress in terms of magnitude and mechanism [60, 61]. Secondly, soybean plants likely adopt varied responses to alkali stress at different growth stages [62]. In leaves, there may be active gene regulation to sustain near-normal levels of photosynthesis and oxidative stress balance under alkaline conditions, potentially leading to a greater number of DEGs. Conversely, during the pod-filling stage, seed genes are predominantly focused on substance accumulation and transformation, showing less sensitivity or alteration in response to alkali stress, resulting in fewer DEGs. Setting up parallel controls in multi-timepoint transcriptome sequencing can better observe whether the DEGs generated under different treatment ratios are induced by different treatments or the result of intrinsic developmental regulation, thus obtaining more information about the interactions [63]. In our experiments, we established parallel controls at various time points to accurately discern the DEGs attributable to specific treatments.

Melatonin functions as a primary defense against oxidative stress and is a crucial component in abiotic stress resistance in plants [64]. Although melatonin receptors have been identified in *Arabidopsis* [23], the intricacies of the signaling pathways they mediate remain to be fully elucidated. Understanding the functional role of melatonin and its regulatory network is a vital avenue for future research. In conclusion, our study employed a combined transcriptomic and metabolomic approach to identify DEGs and DAMs. This analysis elucidated the significant role of melatonin in enhancing alkali stress tolerance mechanisms in soybean, contributing to the construction of comprehensive metabolic regulatory networks. These networks enhance our understanding of the mechanisms underlying plant tolerance to alkali stress.

## Conclusions

In this comprehensive study, we have delineated the mechanisms by which melatonin enhances alkali stress tolerance in soybean, employing integrated transcriptomic and metabolomic analyses. Melatonin application under alkali stress conditions significantly ameliorated the decline in photosynthetic gas exchange parameters, augmented SOD activity, and increased soluble sugar content, effectively scavenging reactive oxygen species. This treatment also reduced malondialdehyde content, indicative of decreased lipid peroxidation, and notably enhanced soybean yield per plant at the pod-filling stage. A total of 183 transcription factors showed differential expression in response to alkali stress with and without melatonin treatment, encompassing key families such as *AP2*/*ERF*, *bHLH*, *MYB*, *WRKY*, *NAC*, and *C2H2*. WGCNA identified pivotal hub genes (*SoyZH13_11G127400*, *SoyZH13_17G200400*, and *SoyZH13_04G073701*) within specific modules, suggesting their involvement in the regulatory mechanisms of melatonin’s response to alkali stress. Furthermore, metabolite and hormone analyses indicated that levels of cytokinins and gibberellins were elevated in the AS + MT group compared to AS treatment. Luteolin-7-O-(2''-O-rhamnosyl)rutinoside and the terpenoid Hederagenin-3-O-glucuronide-28-O-glucosyl(1,2)glucoside emerged as the most highly correlated metabolites, with the latter being regulated by *SoyZH13_04G073701*. This study reveals that melatonin improves soybean yield by systematically regulating hormone levels, transcription factors and expression of resistance genes. It enhances antioxidant enzyme activity and various osmoregulatory substances, sustaining photosynthesis levels and mitigating the inhibitory effects of alkali stress on soybean growth and development. These insights offer valuable contributions to our understanding of the multifaceted role of melatonin in plant stress physiology and provide a foundation for developing strategies to enhance crop resilience to environmental stresses.

### Supplementary Information

Supplementary Material 1.

## Data Availability

The datasets presented in this study can be found in online repositories. The second-generation sequencing data of Zhonghuang13 have been submitted to the NCBI BioProject under accession numbers PRJNA1048376.

## Abbreviations

IAA: Involving Auxin
ABA: Abscisic acid
SA: Salicylic acid
CK: Cytokinin
JA: Jasmonic acid
AP2/ERF: APETALA2/ethylene-responsive factor
NAC: NAM,ATAF1/2,CUC1/2
bHLH: Basic helix-loop-helix
SOD: Superoxide dismutase
MDA: Malondialdehyde
TPM: Transcripts per kilobase of exon model per million mapped reads
DEGs: Differentially expressed genes
FDR: False discovery rate
GO: Gene ontology
KEGG: Kyoto encyclopedia of genes and genomes
UPLC-MS/MS: Ultra-high performance liquid chromatography-tandem mass spectrometry
PCA: Principal component analysis
OPLS-DA: Orthogonal partial least squares discriminant analysis
DAMs: Differentially abundant metabolites
WGCNA: Weighted gene co-expression network analysis
RT-qPCR: Quantitative real-time PCR
ARF: Auxin response factor
AUX/IAA: Auxin/indole-3-acetic acid
GH3: Gretchen Hagen 3
CTK: Cytokinin
GA: Gibberellin
TFs: Transcription factors
EDR1: Enhanced Disease Resistance 1
MAPK: Mitogen-activated protein kinase
CDPK: Ca2^+^-dependent protein kinase
