# Supplementary material for: Elucidating the role of exogenous melatonin in mitigating alkaline stress in soybeans across different growth stages: a transcriptomic and metabolomic approach
Source: BMC Plant Biol. 2024 May 8;24:380. doi: 10.1186/s12870-024-05101-9 (PMC11077714; doi:10.1186/s12870-024-05101-9)
Supplement: Supplementary file 1 — Supplementary Material 1. [file 12870_2024_5101_MOESM1_ESM.docx]

**Supporting Information**

**Methods**

**Plant materials and processing conditions**

The alkali stress treatments were applied every night 9 p.m. at the V3 stage of soybean vegetative growth stage, and the melatonin treatment was carried out at the same time. Sampling of relevant physiological indicators was carried out at 9:00 a.m. on the 4th day after the last treatment, and fully expanded soybean leaves above ground were taken for each treatment for each indicator. Transcriptome sampling was performed at 0h, 3h and 72h after the first application of melatonin.

Alkali stress treatments were carried out 9 p.m. after dark each day at the R5 stage of the soybean pod-filling stages, and melatonin treatments were applied at the same time. all plants were given 100 ml of water in the afternoon in addition to the 9:00 pm treatment to ensure proper plant growth. Sampling of relevant physiological indices was carried out at 9:00 am on the 4th day after the last treatment. Transcriptome sampling was performed at 0h, 3h, 24h, 72h and 10d after the first melatonin application, and full and homogeneous pods were selected for sampling, and hormone metabolite sampling was performed at the 10th day after treatment.

**Transcriptomic sequencing and differential gene expression analysis**

High-quality genomic DNA was extracted from leaf samples at the vegetative growth stage and pod samples at the pod-filling stages of the treatment using the CATB method. The mass of the DNA samples was determined in 0.8% agarose gel electrophoresis. Quantification of DNA concentration using NanoDrop 2000 spectrophotometer (NanoDrop Technologies, Wilmington, DE,USA)，Qubit dsDNA HS Assay Kit on a Qubit 3.0 Fluorometer (Life Technologies, Carlsbad, CA, USA). Construct sequencing libraries using VAHTS Universal DNA Library Prep Kit for MGI (Vazyme, Nanjing, China) and add index codes to the sequences of the samples. The size and quantification of libraries were assessed using Qubit 3.0 Fluorometer (Life Technologies, Carlsbad, CA, USA) and Bioanalyzer 2100 system (Agilent Technologies, CA, USA). Subsequently, sequencing was performed on MGISEQ-T7 platform by Frasergen Bioinformatics Co., Ltd.

**Metabolomic analysis by ultra-high performance liquid chromatography-tandem mass spectrometry (UPLC-MS/MS)**

Twelve samples were processed for metabolomics analysis by ultra-high performance liquid chromatography/tandem mass spectrometry (UPLC-MS/MS). The freeze-dried samples were placed in a zirconia bead mixer pulveriser (MM 400, Retsch) with the parameters set to 30 Hz,1.5 min. Then 100 mg of the lyophilised powder was solubilised with 1.2 ml of 70% methanol solution, and the treated samples were placed in the refrigerator at 4°C for overnight precipitation, centrifuged at 12000 rpm for 10 min (4°C), and the extracts were filtered (SCAA-104, 0.22 μm pore size; ANPEL, Shanghai, China) and used for subsequent UPLC- MS/MS analysis.

All samples were analysed using a UPLC-ESI-MS/MS system (UPLC, SHIMADZU Nexera X2; MS, Applied Biosystems 4500 Q TRAP) following machine instructions. Agilent SB-C18 (1.8 µm,2.1 mm * 100 mm), The column temperature was maintained at 40 °C, the flow rate was 0.35 mL/min and the mobile phase consisted of solvent A (pure water with 0.1% formic acid) and solvent B (acetonitrile with 0.1% formic acid). The gradient elution conditions were set as follows: Sample measurements were performed with a gradient program that employed the starting conditions of 95% A, 5% B. Within 9 min, a linear gradient to 5% A, 95% B was programmed, and a composition of 5% A, 95% B was kept for 1 min. Subsequently, a composition of 95% A, 5.0% B was adjusted within 1.1 min and kept for 2.9 min. The injection volume of each sample was 4 μL.

Triple quadrupole-linear ion trap mass spectrometer (Q TRAP) for the detection of metabolites eluting from the column, Controlled by Analyst 1.6.3 software (AB Sciex) running both positive and negative ionisation modes. The system is equipped with the ESI Turbo Ion Spray Interface, The ESI source operating parameters are as follows: source temperature 550°C; ion spray voltage (IS) 5500 V (positive ion mode)/-4500 V (negative ion mode); ion source gas I (GSI), gas II(GSII), curtain gas (CUR) were set at 50, 60, and 25.0 psi, respectively; the collision-activated dissociation (CAD) was high. Instrument tuning and mass calibration were performed with 10 and 100 μmol/L polypropylene glycol solutions in QQQ and LIT modes, respectively. QQQ scans were acquired as MRM experiments with collision gas (nitrogen) set to medium. In order to make the quantification more precise and stable, a quality control sample (a collection of all samples) was taken after every 10 samples. Peak extraction is mainly achieved through the MultiaQuant software.

**Table S1** **Information on qRT-PCR primers**

| Primer name | Primer sequence | TM value（℃） | | Product length （bp） |
| --- | --- | --- | --- | --- |
| *SoyZH13_05G222200*-F | AAACCCCTTACCCTCGTGTG | | 59.60 | 84 |
| *SoyZH13_05G222200*-R | AGGAATAAGAAGGGGGAGCA | | 57.71 |  |
| *SoyZH13_19G128500*-F | GGCAAAACTTGGAGACTGTGC | | 60.27 | 112 |
| *SoyZH13_19G128500*-R  *SoyZH13_CG010200*-F  *SoyZH13_CG010200*-R  *SoyZH13_12G159300*-F  *SoyZH13_12G159300*-R  *SoyZH13_05G208600*-F  *SoyZH13_05G208600*-R  *SoyZH13_CG005300*-F  *SoyZH13_CG005300*-R  *SoyZH13_15G161700*-F  *SoyZH13_15G161700*-R  *SoyZH13_02G052101*-F  *SoyZH13_02G052101*-R  *SoyZH13_12G047500*-F  *SoyZH13_12G047500*-R  *SoyZH13_02G085500*-F  *SoyZH13_02G085500*-R  *SoyZH13_08G174000*-F  *SoyZH13_08G174000*-R  *SoyZH13_09G211300*-F  *SoyZH13_09G211300*-R  *SoyZH13_11G026800*-F  *SoyZH13_11G026800*-R  *SoyZH13_10G057000*-F  *SoyZH13_10G057000*-R  *SoyZH13_06G291900*-F  *SoyZH13_06G291900*-R  *SoyZH13_13G004200*-F  *SoyZH13_13G004200*-R  *SoyZH13_01G053101*-F  *SoyZH13_01G053101*-R  *SoyZH13_20G025001*-F  *SoyZH13_20G025001*-R  *SoyZH13_13G173200*-F  *SoyZH13_13G173200*-R  *GmACT*-F  *GmACT*-R  *GmCYP2*-F  *GmCYP2*-R  *GmELF1B*-F  *GmELF1B*-R | CATTTGCGATGTGGGTGCAA  CAACTACTTATTTATGTGGGCGCT  TCCCCAACAGTCCAAAGACG  TCCCGCCACTACCAAGAAAG  GTTGGGCCGTTGTCTTGTTG  TTGTCGTTGCTAGGGCGTAT  AGCAGAAGGGGATGGTTTGT  ATGGCCGATACTACTGGAAGGA  CTACAGAGATGAGCCCAATCCG  GGCATCTTGTGTTGTGTCCC  AGGCTCTGTCAAGGGAGAGT  CCCCTGACAGATGGCAGAAT  GGCCATGTTCAATGCGTTTG  CAGAGTTGCACGCCTCTACC  GGTCTCCTTTTGGGTCTGCC  TCTTGATCTGGCCGGTCGT  CTCGTAGCTCTTCTCGACGG  GTGAGCCACACAGTTCCCA  TCAAGGGCCACATATGCCAA  TGTGCAGCATCAGTGAGAAAG  CCCTCCGGGAAGATGAGAGA  GGTTGTCCGAGGCAAGTGT  ACCGCCAGACCAAAGAACTC  TCCATTGCGAGCTACGTCTC  GCTGGCAATCTCTGAAGGGT  GGTGGTGTCGGTGTTAGAGC  GTGTGTGAGTCCGTTCTGCT  CGGCACTTCTTCGTCATAGGG  CCCCACTGGAGTAGTTAATGC  CAGTGGCTCTATGGAAACGGG  GGACCTCGTTCTTCTGCTCTT  GGAAGCCGAACTGCCATAGA  CCGAGGCTGCATAGTTAGGG  ATGCAAGAAGGGACAGGCAG  GACAATCACTTGAGCGGCAC  GTGAGCCACCAGTTCCCAT  TAAATCACGGCCAGCAAGGT  CAAAAACCCTGTCACGCAGT  CACTTTCTCTCAAGGGCACCA  TTCTCAGATCTACACACCGAAG  TTGTCAACTGATCCCCAGAAAT | | 60.04  59.43  59.89  59.68  60.25  59.47  59.22  60.43  60.29  59.40  59.88  59.45  58.93  60.74  60.61  60.98  59.70  59.85  59.67  58.85  60.11  60.23  60.25  59.90  60.03  60.67  60.25  60.80  58.08  60.74  59.73  59.82  59.97  60.23  59.83  83.5  83.3  84  83.8  82  81.9 | 107  96  198  123  280  108  91  193  191  261  141  204  150  248  87  118  202  81  255  88 |

Note：Sequence ID name corresponding to the housekeeping genes：*GmACT-soyZH13_08G174000*, *GmCYP2-soyZH13_12G024001*, *GmELF1B- soyZH13_02G254500*.

**Table S2** Plant height and leaf area at vegetative growth stage

|  | CK | AS | AS+MT | MT |
| --- | --- | --- | --- | --- |
| Plant height （cm） | 20.98±1.34b | 18.30±0.51c | 20.72±1.07b | 23.14±1.11a |
| Leaf area（mm^2^） | 2153.88±42.51b | 1799.77±60.28d | 1993.74±43.02c | 2383.52±64.67a |

Note: Distinct letters (a, b, c, d) accompanying the results for each treatment denote significant differences at the 0.05 level. CK refers to the control treatment; AS indicates the alkalizing salt stress treatment; AS+MT represents the combined alkalizing salt and melatonin treatment; and MT stands for the melatonin treatment alone. Post hoc multiple comparisons were conducted using the Least Significant Difference (LSD) method.

**Table S3** Statistics of RNA sequencing data for soybean at vegetative growth stage

| Sample Name | Primitive base （Gb） | Clean base （Gb） | Q20 Base ratio（%） | Q30 Base ratio（%） | GC content  （%） |
| --- | --- | --- | --- | --- | --- |
| **V3_CK_0 h_1** | 6.68 | 5.94 | 98.79 | 94.04 | 42.99 |
| **V3_CK_0 h_2** | 6.73 | 5.94 | 98.68 | 93.53 | 42.14 |
| **V3_CK_0 h_3** | 6.76 | 5.93 | 98.58 | 93.19 | 43.05 |
| **V3_CK_3 h_1** | 7.21 | 6.45 | 98.31 | 94.36 | 44.58 |
| **V3_CK_3 h_2** | 7.29 | 6.49 | 98.52 | 92.99 | 44.46 |
| **V3_CK_3 h_3** | 7.68 | 6.77 | 98.35 | 92.36 | 44.69 |
| **V3_CK_72 h_1** | 7.35 | 5.49 | 97.19 | 90.65 | 46.12 |
| **V3_CK_72 h_2** | 7.70 | 6.67 | 97.83 | 91.13 | 45.92 |
| **V3_CK_72 h_3** | 6.52 | 5.70 | 97.80 | 91.10 | 45.23 |
| **V3_AS_3 h_1** | 6.86 | 6.09 | 98.42 | 92.62 | 44.38 |
| **V3_AS_3 h_2** | 8.16 | 7.22 | 98.39 | 92.48 | 44.97 |
| **V3_AS_3 h_3** | 6.70 | 5.90 | 98.39 | 92.36 | 44.03 |
| **V3_AS_72 h_1** | 6.17 | 5.33 | 97.75 | 91.09 | 44.64 |
| **V3_AS_72 h_2** | 7.88 | 6.84 | 97.82 | 91.12 | 46.01 |
| **V3_AS_72 h_3** | 7.88 | 6.83 | 97.84 | 91.18 | 45.82 |
| **V3_AS+MT_3 h_1** | 7.68 | 5.85 | 97.17 | 95.50 | 44.99 |
| **V3_AS+MT_3 h_2** | 7.47 | 6.51 | 97.88 | 91.29 | 45.24 |
| **V3_AS+MT_3 h_3** | 6.80 | 5.87 | 97.74 | 90.91 | 44.75 |
| **V3_AS+MT_72 h_1** | 6.67 | 5.87 | 98.35 | 92.24 | 43.38 |
| **V3_AS+MT_72 h_2** | 6.52 | 5.68 | 97.95 | 91.62 | 46.02 |
| **V3_AS+MT_72 h_3** | 6.98 | 6.05 | 98.01 | 91.77 | 44.18 |
| **V3_MT_3 h_1** | 6.69 | 5.94 | 98.57 | 93.04 | 43.80 |
| **V3_MT_3 h_2** | 6.36 | 5.67 | 98.60 | 93.19 | 43.82 |
| **V3_MT_3 h_3** | 6.02 | 5.37 | 98.48 | 92.85 | 43.79 |
| **V3_MT_72 h_1** | 6.83 | 5.99 | 98.31 | 92.05 | 43.87 |
| **V3_MT_72 h_2** | 7.23 | 6.34 | 98.34 | 92.16 | 42.96 |
| **V3_MT_72 h_3** | 7.53 | 6.61 | 98.30 | 92.01 | 43.37 |

**Table S4** Statistics of RNA sequencing data for soybean at the pod-filling stage

| Sample Name | Primitive base （Gb） | Clean base （Gb） | Q20 Base ratio（%） | Q30 Base ratio（%） | GC content  （%） |
| --- | --- | --- | --- | --- | --- |
| **R5_CK_0 h_1** | 7.13 | 6.23 | 98.23 | 91.94 | 46.67 |
| **R5_CK_0 h_2** | 6.37 | 5.55 | 98.21 | 91.25 | 46.80 |
| **R5_CK_0 h_3** | 7.10 | 6.17 | 97.95 | 90.92 | 46.32 |
| **R5_CK_0 h_4** | 7.52 | 6.52 | 98.03 | 91.19 | 46.93 |
| **R5_CK_0 h_5** | 6.48 | 5.66 | 98.17 | 91.71 | 46.20 |
| **R5_CK_3 h_1** | 6.15 | 5.33 | 98.03 | 91.19 | 46.41 |
| **R5_CK_3 h_2** | 6.44 | 5.72 | 98.46 | 92.80 | 46.21 |
| **R5_CK_3 h_3** | 7.39 | 6.51 | 98.38 | 92.47 | 46.38 |
| **R5_CK_24 h_1** | 8.34 | 7.26 | 98.20 | 91.80 | 46.49 |
| **R5_CK_24 h_2** | 8.12 | 7.09 | 98.18 | 91.88 | 46.68 |
| **R5_CK_24 h_3** | 7.85 | 6.85 | 98.22 | 92.02 | 46.60 |
| **R5_CK_72 h_1** | 6.80 | 5.83 | 98.12 | 92.13 | 45.98 |
| **R5_CK_72 h_2** | 6.66 | 5.66 | 98.04 | 92.02 | 46.55 |
| **R5_CK_72 h_3** | 6.65 | 5.69 | 98.08 | 92.03 | 46.36 |
| **R5_CK_10 d_1** | 6.28 | 5.15 | 97.70 | 91.04 | 46.64 |
| **R5_CK_10 d_2** | 6.78 | 5.70 | 98.05 | 91.98 | 45.97 |
| **R5_CK_10 d_3** | 6.57 | 5.45 | 97.83 | 91.27 | 46.49 |
| **R5_AS_3 h_1** | 6.89 | 6.09 | 98.43 | 92.68 | 46.41 |
| **R5_AS_3 h_2** | 7.10 | 6.25 | 98.44 | 92.67 | 46.33 |
| **R5_AS_3 h_3** | 7.16 | 6.30 | 98.33 | 92.29 | 46.24 |
| **R5_AS_24h_1** | 7.84 | 6.89 | 98.30 | 92.31 | 46.03 |
| **R5_AS_24h_2** | 8.26 | 7.24 | 98.13 | 91.69 | 45.62 |
| **R5_AS_24h_3** | 7.41 | 6.45 | 98.25 | 92.16 | 47.08 |
| **R5_AS_72 h_1** | 6.27 | 5.29 | 97.82 | 91.30 | 43.31 |
| **R5_AS_72 h_2** | 9.57 | 8.17 | 98.23 | 92.09 | 46.47 |
| **R5_AS_72 h_3** | 6.35 | 5.24 | 97.72 | 91.02 | 46.42 |
| **R5_AS_10 d_1** | 6.59 | 5.54 | 98.09 | 92.10 | 46.28 |
| **R5_AS_10 d_2** | 6.56 | 5.37 | 97.88 | 91.91 | 46.76 |
| **R5_AS_10 d_3** | 6.53 | 5.35 | 97.74 | 91.26 | 47.14 |
| **R5_AS+MT_3 h_1** | 7.08 | 6.22 | 98.32 | 92.23 | 46.38 |
| **R5_AS+MT_3 h_2** | 7.52 | 6.59 | 98.32 | 92.26 | 46.31 |
| **R5_AS+MT_3 h_3** | 7.70 | 6.77 | 98.32 | 92.23 | 46.53 |
| **R5_AS+MT_24 h_1** | 6.38 | 5.46 | 98.07 | 92.07 | 46.67 |
| **R5_AS+MT_24 h_2** | 6.46 | 5.42 | 97.74 | 91.06 | 46.36 |
| **R5_AS+MT_24 h_3** | 6.55 | 5.58 | 98.05 | 91.98 | 46.51 |
| **R5_AS+MT_72 h_1** | 6.32 | 5.22 | 97.81 | 91.49 | 47.30 |
| **R5_AS+MT_72 h_2** | 6.53 | 5.53 | 98.15 | 92.39 | 46.33 |
| **R5_AS+MT_72 h_3** | 6.89 | 5.68 | 98.03 | 92.02 | 46.64 |
| **R5_AS+MT_10 d_1** | 6.50 | 5.35 | 97.73 | 91.03 | 46.63 |
| **R5_AS+MT_10 d_2** | 6.55 | 5.45 | 97.76 | 91.02 | 46.36 |
| **R5_AS+MT_10 d_3** | 6.03 | 4.49 | 97.65 | 90.89 | 47.11 |
| **R5_MT_3 h_1** | 6.97 | 6.06 | 98.21 | 91.85 | 46.05 |
| **R5_MT_3 h_2** | 7.74 | 6.82 | 98.34 | 92.33 | 46.76 |
| **R5_MT_3 h_3** | 7.33 | 6.42 | 98.30 | 92.18 | 46.63 |
| **R5_MT_24 h_1** | 6.61 | 5.61 | 97.93 | 91.48 | 46.00 |
| **R5_MT_24 h_2** | 6.27 | 5.23 | 97.70 | 91.05 | 46.69 |
| **R5_MT_24 h_3** | 6.18 | 5.17 | 97.79 | 91.28 | 46.38 |
| **R5_MT_72 h_1** | 6.16 | 5.05 | 97.76 | 91.39 | 46.78 |
| **R5_MT_72 h_2** | 6.38 | 5.27 | 97.79 | 91.21 | 47.13 |
| **R5_MT_72 h_3** | 6.86 | 5.60 | 97.68 | 91.00 | 46.12 |
| **R5_MT_10 d_1** | 7.18 | 6.39 | 98.68 | 92.57 | 46.47 |
| **R5_MT_10 d_2** | 7.15 | 6.30 | 98.51 | 92.84 | 47.00 |
| **R5_MT_10 d_3** | 7.02 | 6.25 | 98.65 | 93.44 | 46.89 |

**Table S5** Differential metabolites under AS treatment and AS+MT treatment

| Index | Compounds | | J-1 | | J-2 | | J-3 | | JM-1 | | JM-2 | | JM-3 | | Fold_Change | | Type | |  |  |  |
| --- | --- | --- | --- | --- | --- | --- | --- | --- | --- | --- | --- | --- | --- | --- | --- | --- | --- | --- | --- | --- | --- |
| mws0576 | 3-Hydroxybutyric acid | | 1.70E+06 | | 1.43E+06 | | 1.37E+06 | | 9.00E+00 | | 9.00E+00 | | 9.00E+00 | | 5.99E-06 | | down | |  |  |  |
| mws0262 | L-Tartaric acid | | 3.77E+04 | | 4.04E+04 | | 5.57E+04 | | 1.04E+05 | | 7.40E+04 | | 1.09E+05 | | 2.14E+00 | | up | |  |  |  |
| Lmbp000123 | L-Homomethionine | | 6.93E+05 | | 3.72E+05 | | 6.68E+05 | | 2.45E+05 | | 1.28E+05 | | 3.94E+05 | | 4.43E-01 | | down | |  |  |  |
| mws0851 | Sodium Valproate | | 1.16E+06 | | 1.01E+06 | | 7.21E+05 | | 3.04E+05 | | 2.60E+05 | | 1.85E+05 | | 2.59E-01 | | down | |  |  |  |
| Cmdn000784 | Uric acid | | 8.17E+03 | | 6.12E+03 | | 6.81E+03 | | 9.49E+03 | | 1.19E+04 | | 2.42E+04 | | 2.16E+00 | | up | |  |  |  |
| mws1155 | D-Mannitol* | | 8.54E+03 | | 5.33E+03 | | 1.71E+04 | | 1.60E+04 | | 3.52E+04 | | 1.41E+04 | | 2.11E+00 | | up | |  |  |  |
| pme3882 | 2'-Deoxyuridine | | 2.49E+04 | | 1.96E+04 | | 2.35E+04 | | 9.06E+03 | | 4.53E+03 | | 1.79E+04 | | 4.63E-01 | | down | |  |  |  |
| Zmjn003398 | 2-Dodecenedioic acid | | 1.31E+04 | | 1.84E+04 | | 2.41E+04 | | 2.68E+04 | | 3.57E+04 | | 6.48E+04 | | 2.29E+00 | | up | |  |  |  |
| Hmlp001371 | Cyclo(Tyr-Ala) | | 1.21E+04 | | 1.55E+04 | | 8.59E+03 | | 2.89E+03 | | 6.91E+03 | | 5.24E+03 | | 4.15E-01 | | down | |  |  |  |
| pmb0962 | L-Lysine-Butanoic Acid | | 2.82E+05 | | 1.05E+05 | | 1.65E+05 | | 4.53E+04 | | 9.61E+04 | | 1.04E+05 | | 4.46E-01 | | down | |  |  |  |
| pme0264 | Thymidine | | 4.90E+05 | | 3.77E+05 | | 3.62E+05 | | 1.67E+05 | | 1.43E+05 | | 2.93E+05 | | 4.91E-01 | | down | |  |  |  |
| MWS2430 | 13-methylmyristic acid | | 1.91E+05 | | 1.61E+05 | | 1.91E+05 | | 7.39E+04 | | 8.30E+04 | | 1.02E+05 | | 4.75E-01 | | down | |  |  |  |
| Index | Compounds | | J-1 | | J-2 | | J-3 | | JM-1 | | JM-2 | | JM-3 | | Fold_Change | | Type | |  |  |  |
| pme2563 | γ-Glu-Cys | | 2.76E+04 | | 1.87E+04 | | 2.31E+04 | | 8.23E+04 | | 6.08E+04 | | 4.04E+04 | | 2.64E+00 | | up | |  |  |  |
| mws1488 | Palmitic acid | | 8.74E+04 | | 1.06E+05 | | 8.93E+04 | | 5.50E+04 | | 3.38E+04 | | 4.70E+04 | | 4.81E-01 | | down | |  |  |  |
| pmb0952 | Thiamine (Vitamin B1) | | 1.17E+04 | | 1.92E+04 | | 5.07E+04 | | 7.60E+04 | | 4.58E+04 | | 4.76E+04 | | 2.07E+00 | | up | |  |  |  |
| mws0383 | 10-Heptadecenoic Acid | | 1.31E+06 | | 8.84E+05 | | 7.96E+05 | | 2.96E+05 | | 1.87E+05 | | 4.38E+05 | | 3.08E-01 | | down | |  |  |  |
| Lmyn006227 | Galangin (3,5,7-Trihydroxyflavone) | | 5.13E+04 | | 4.10E+04 | | 4.34E+04 | | 8.71E+04 | | 1.38E+05 | | 7.77E+04 | | 2.23E+00 | | up | |  |  |  |
| YC512118 | Oleamide (9-Octadecenamide) | | 6.95E+03 | | 4.95E+03 | | 6.75E+03 | | 2.20E+03 | | 2.61E+03 | | 4.29E+03 | | 4.88E-01 | | down | |  |  |  |
| mws2623 | 11-Octadecanoic acid(Vaccenic acid) | | 7.69E+07 | | 6.00E+07 | | 6.28E+07 | | 2.25E+07 | | 2.12E+07 | | 2.95E+07 | | 3.66E-01 | | down | |  |  |  |
| Lmyn012331 | Petroselinic acid | | 7.58E+07 | | 6.04E+07 | | 6.48E+07 | | 2.11E+07 | | 2.01E+07 | | 2.92E+07 | | 3.50E-01 | | down | |  |  |  |
| mws1489 | Stearic Acid | | 5.30E+06 | | 5.13E+06 | | 4.93E+06 | | 2.21E+06 | | 2.68E+06 | | 2.76E+06 | | 4.98E-01 | | down | |  |  |  |
| Lmcn009539 | E,E,Z-1,3,12-Nonadecatriene-5,14-diol | | 7.92E+04 | | 5.91E+04 | | 5.14E+04 | | 2.40E+04 | | 1.93E+04 | | 3.37E+04 | | 4.06E-01 | | down | |  |  |  |
| pmb3079 | N-Acetyl-D-glucosamine-1-phosphate | | 8.87E+04 | | 3.31E+04 | | 5.17E+04 | | 9.00E+00 | | 9.00E+00 | | 9.00E+00 | | 1.56E-04 | | down | |  |  |  |
| Cmmn013275 | Isopimaric acid | | 6.36E+04 | | 8.25E+04 | | 9.01E+04 | | 3.08E+04 | | 3.19E+04 | | 4.79E+04 | | 4.68E-01 | | down | |  |  |  |
| mws0042 | Epigallocatechin | | 1.29E+04 | | 9.81E+03 | | 6.58E+03 | | 1.59E+04 | | 3.00E+04 | | 2.85E+04 | | 2.54E+00 | | up | |  |  |  |
| pmn001610 | Eicosadienoic acid | | 4.65E+05 | | 1.73E+05 | | 1.80E+05 | | 4.40E+04 | | 4.92E+04 | | 5.58E+04 | | 1.82E-01 | | down | |  |  |  |
| Lmhn003074 | Feruloylmalic acid | | 3.29E+04 | | 4.96E+04 | | 3.20E+04 | | 8.80E+04 | | 6.99E+04 | | 8.07E+04 | | 2.08E+00 | | up | |  |  |  |
| pmn001606 | Eicosenoic acid | | 1.96E+06 | | 1.78E+06 | | 8.77E+05 | | 7.14E+05 | | 8.61E+05 | | 6.11E+05 | | 4.73E-01 | | down | |  |  |  |
| pmn001518 | 1-O-Galloyl-D-glucose* | | 1.87E+05 | | 1.80E+05 | | 1.78E+05 | | 9.02E+04 | | 7.87E+04 | | 9.67E+04 | | 4.87E-01 | | down | |  |  |  |
| Hmln000873 | 2-O-Galloyl-D-glucose* | | 1.82E+05 | | 1.73E+05 | | 1.80E+05 | | 9.02E+04 | | 9.17E+04 | | 8.43E+04 | | 4.97E-01 | | down | |  |  |  |
| pmn001421 | | 3-O-p-Coumaroylquinic acid* | | 9.00E+00 | | 9.00E+00 | | 9.00E+00 | | 2.59E+04 | | 2.30E+04 | | 1.26E+04 | | 2.28E+03 | | up | |  |  |
| pmb3074 | | 5-O-p-Coumaroylquinic acid* | | 9.00E+00 | | 9.00E+00 | | 9.00E+00 | | 2.31E+04 | | 2.28E+04 | | 1.51E+04 | | 2.26E+03 | | up | |  |  |
| pmb0296 | | 1-Oleoyl-Sn-Glycerol | | 6.96E+04 | | 7.71E+04 | | 6.73E+04 | | 2.32E+04 | | 3.23E+04 | | 3.69E+04 | | 4.32E-01 | | down | |  |  |
| Lmmp010562 | | Diisooctyl Phthalate | | 9.59E+05 | | 1.01E+06 | | 7.18E+05 | | 4.73E+05 | | 3.82E+05 | | 4.60E+05 | | 4.89E-01 | | down | |  |  |
| Lmwp011196 | | Bis(2-ethylhexyl)phthalate | | 1.15E+06 | | 1.09E+06 | | 7.44E+05 | | 4.86E+05 | | 4.05E+05 | | 4.96E+05 | | 4.65E-01 | | down | |  |  |
| Lmbp003668 | | Kaempferol-3-O-arabinoside | | 1.07E+04 | | 2.10E+04 | | 2.02E+04 | | 1.07E+05 | | 7.73E+04 | | 1.80E+04 | | 3.89E+00 | | up | |  |  |
| mws1089 | | Sucrose-6-phosphate | | 7.01E+04 | | 3.88E+04 | | 4.66E+04 | | 9.00E+00 | | 9.00E+00 | | 9.00E+00 | | 1.74E-04 | | down | |  |  |
| Lmfn004093 | | Phloretin-4'-O-glucoside (Trilobatin) | | 1.10E+04 | | 2.63E+04 | | 3.43E+04 | | 3.95E+04 | | 1.55E+05 | | 6.13E+04 | | 3.57E+00 | | up | |  |  |
| MWSHY0031 | | Apigenin-7-O-glucuronide | | 8.69E+03 | | 1.97E+04 | | 2.45E+04 | | 2.11E+04 | | 3.38E+04 | | 1.10E+05 | | 3.11E+00 | | up | |  |  |
| MWSHY0104 | | Luteolin-7-O-glucoside (Cynaroside)* | | 7.86E+05 | | 9.93E+05 | | 1.37E+06 | | 3.99E+06 | | 2.63E+06 | | 1.13E+06 | | 2.46E+00 | | up | |  |  |
| pmb2654 | | Anthranilate-1-O-Sophoroside | | 1.09E+07 | | 1.05E+07 | | 1.26E+07 | | 3.49E+06 | | 5.81E+06 | | 7.52E+06 | | 4.95E-01 | | down | |  |  |
| mws4167 | | Luteolin-7-O-glucuronide | | 3.22E+03 | | 1.05E+04 | | 7.30E+03 | | 7.25E+03 | | 1.36E+04 | | 2.17E+04 | | 2.02E+00 | | up | |  |  |
| pme1683 | | Calcium Pantothenate | | 2.64E+04 | | 3.47E+04 | | 3.69E+04 | | 1.05E+04 | | 1.67E+04 | | 1.42E+04 | | 4.23E-01 | | down | |  |  |
| Lmjp003295 | | 6-Methoxykaempferol-3-O-glucoside | | 1.50E+06 | | 8.69E+05 | | 1.15E+06 | | 4.10E+05 | | 2.96E+05 | | 6.93E+05 | | 3.98E-01 | | down | |  |  |
| Lmjp002906 | | Rhamnetin-3-O-Glucoside* | | 8.18E+05 | | 5.33E+05 | | 5.27E+05 | | 9.71E+04 | | 1.61E+05 | | 5.47E+05 | | 4.29E-01 | | down | |  |  |
| Lmjp003044 | | Isorhamnetin-3-O-Glucoside* | | 7.87E+05 | | 5.20E+05 | | 5.36E+05 | | 7.68E+04 | | 1.32E+05 | | 5.81E+05 | | 4.29E-01 | | down | |  |  |
| Hmcp002207 | | Isorhamnetin-7-O-glucoside (Brassicin)* | | 7.83E+05 | | 5.33E+05 | | 5.44E+05 | | 5.87E+04 | | 1.59E+05 | | 5.09E+05 | | 3.91E-01 | | down | |  |  |
| pmb2975 | | Hesperetin-3'-O-glucuronide | | 1.45E+04 | | 1.03E+04 | | 1.43E+04 | | 2.34E+03 | | 7.49E+03 | | 6.38E+03 | | 4.14E-01 | | down | |  |  |
| Lmmn003398 | | Kaempferol-3-O-(6''-acetyl)glucoside | | 5.85E+05 | | 7.65E+05 | | 1.02E+06 | | 1.11E+05 | | 1.35E+05 | | 1.07E+05 | | 1.49E-01 | | down | |  |  |
| Hmbn002692 | | 6'-O-Feruloyl-D-sucrose | | 9.88E+04 | | 5.90E+04 | | 7.53E+04 | | 2.99E+04 | | 4.38E+04 | | 4.23E+04 | | 4.97E-01 | | down | |  |  |
| Li512111 | | | | Isorhamnetin-3-O-(6''-acetylglucoside) | | 2.88E+04 | | 1.07E+04 | | 1.14E+04 | | 6.95E+03 | | 3.80E+03 | | 1.08E+04 | | 4.22E-01 | | down | |
| Lmdp004892 | | | Kaempferol-3-O-(6''-malonyl)galactoside* | | 1.22E+06 | | 1.59E+06 | | 1.89E+06 | | 3.27E+05 | | 3.00E+05 | | 1.06E+06 | | 3.59E-01 | | down | |  |
| HJAP064 | | | Isorhamnetin-3-O-(6''-malonylglucoside) | | 6.71E+04 | | 5.54E+04 | | 6.38E+04 | | 6.05E+03 | | 1.74E+04 | | 5.74E+04 | | 4.34E-01 | | down | |  |
| Lmmp002963 | | 6-C-Methylquercetin-3-O-rutinoside | | 1.26E+04 | | 1.58E+04 | | 2.90E+04 | | 4.79E+05 | | 4.46E+05 | | 6.18E+04 | | 1.72E+01 | | up | |  |  |
| Lmhp002800 | | 2'-Hydoxy,5-methoxyGenistein-4',7-O-diglucoside | | 1.17E+05 | | 1.29E+05 | | 1.12E+05 | | 3.85E+04 | | 3.08E+04 | | 8.59E+04 | | 4.34E-01 | | down | |  |  |
| pmp001271 | | 1-Linoleoyl-sn-glycerol-diglucoside | | 6.45E+03 | | 9.46E+03 | | 1.01E+04 | | 2.67E+03 | | 1.94E+03 | | 6.36E+03 | | 4.22E-01 | | down | |  |  |
| Hmcp001629 | | Kaempferol-3-O-(6''-Malonyl)glucoside-7-O-Glucoside | | 1.65E+05 | | 1.80E+05 | | 2.05E+05 | | 3.36E+04 | | 6.90E+04 | | 1.53E+05 | | 4.66E-01 | | down | |  |  |
| Zmlp003063 | | Luteolin-7-O-(2''-O-rhamnosyl)rutinoside | | 9.00E+00 | | 9.00E+00 | | 9.00E+00 | | 4.30E+04 | | 3.16E+04 | | 3.86E+03 | | 2.91E+03 | | up | |  |  |
| Hmdp103165 | | Clitorin | | 9.00E+00 | | 9.00E+00 | | 9.00E+00 | | 2.10E+06 | | 1.67E+06 | | 2.66E+05 | | 1.49E+05 | | up | |  |  |
| Lmpp003268 | | Kaempferol-3-O-rutinoside-7-O-glucoside | | 3.62E+05 | | 2.94E+05 | | 6.92E+05 | | 1.06E+06 | | 1.24E+06 | | 1.18E+06 | | 2.58E+00 | | up | |  |  |
| pmp001105 | | Kaempferol-3-O-neohesperidoside-7-O-glucoside | | 2.78E+05 | | 2.96E+05 | | 6.55E+05 | | 1.15E+06 | | 1.09E+06 | | 1.19E+06 | | 2.80E+00 | | up | |  |  |
| Hmyp008441 | | PE(16:0/18:3+O3) | | 5.10E+03 | | 3.72E+03 | | 2.59E+03 | | 1.71E+04 | | 1.55E+04 | | 6.10E+03 | | 3.39E+00 | | up | |  |  |
| pmn001505 | | Oleanolic acid-3-O-xylosyl(1→3)glucuronide | | 3.28E+04 | | 2.57E+04 | | 4.07E+04 | | 9.00E+00 | | 9.00E+00 | | 9.00E+00 | | 2.72E-04 | | down | |  |  |
| Lmmp006607 | | Hederagenin-3-O-glucuronide-28-O-glucosyl(1,2)glucoside | | 2.06E+04 | | 2.45E+04 | | 1.76E+04 | | 1.75E+05 | | 1.35E+05 | | 3.64E+04 | | 5.53E+00 | | up | |  |  |

**Table S6** Contents of 39 phytohormone metabolites in different treatments (ng/g)

| Index | Compounds | CK | AS | AS+MT | MT |
| --- | --- | --- | --- | --- | --- |
| ABA-GE | ABA-glucosyl ester | 40.06271 | 51.52778 | 65.35293 | 50.58660 |
| ABA | Abscisic acid | 2398.50867 | 3248.16845 | 3576.53854 | 3549.74799 |
| IA | 3-Indoleacrylic acid | 0.70083 | 0.86779 | 1.23455 | 1.32345 |
| IAA-Glu | Indole-3-acetyl glutamic acid | 42.71921 | 75.26202 | 30.57040 | 61.16633 |
| IAA-Phe-Me | Indole-3-acetyl-L-phenylalanne methyle ester | 0.88790 | 0.88891 | 0.91092 | 1.02759 |
| IAA-Trp | Indole-3-acetyl-L-tryptophan | 7.05093 | 11.77483 | 7.63706 | 12.81455 |
| IAA-Glc | 1-O-indol-3-ylacetylglucose | 19.64964 | 22.97141 | 22.52968 | 25.32145 |
| TRP | L-tryptophan | 13825.74858 | 24188.24341 | 17437.39014 | 24383.87847 |
| IAA | Indole-3-acetic acid | 22.04614 | 32.10064 | 32.30641 | 24.30891 |
| OxIAA | 2-oxindole-3-acetic acid | 14.21705 | 17.31924 | 16.15946 | 15.24852 |
| IAA-Asp | Indole-3-acetyl-L-aspartic acid | 55.89201 | 70.77798 | 44.10943 | 71.67091 |
| MEIAA | Methyl indole-3-acetate | 1.56777 | 2.39010 | 3.24561 | 2.12491 |
| IAN | 3-Indoleacetonitrile | 0.08269 | 0.06697 | 0.10220 | 0.13295 |
| ICAld | Indole-3-carboxaldehyde | 46.02304 | 46.42682 | 44.78221 | 53.75462 |
| 2MeScZR | 2-Methylthio-cis-zeatin riboside | 0.01731 | 0.01923 | 0.01941 | 0.01849 |
| tZOG | trans-Zeatin-O-glucoside | 0.38795 | 0.50169 | 0.37045 | 0.47811 |
| IP | N6-isopentenyladenine | 0.02807 | 0.02155 | 0.01334 | 0.02575 |
| cZROG | cis-Zeatin-O-glucoside riboside | 0.06263 | 0.08839 | 0.11306 | 0.10214 |
| cZ9G | cis-Zeatin-9-glucoside | 0.04824 | 0.04339 | 0.05080 | 0.07160 |
| IPR | N6-isopentenyladenosine | 0.02415 | 0.02683 | 0.01957 | 0.03154 |
| DHZROG | Dihydrozeatin-O-glucoside riboside | 0.01625 | 0.04304 | 0.01671 | 0.01935 |
| 2MeSiP | 2-Methylthio-N6-isopentenyladenine | 0.05186 | 0.00000 | 0.00000 | 0.00000 |
| oT | ortho-Topolin | 0.08899 | 0.01651 | 0.07097 | 0.00000 |
| DHZR | Dihydrozeatin ribonucleoside | 0.12967 | 0.09531 | 0.12660 | 0.09397 |
| tZ | trans-Zeatin | 0.20345 | 0.20419 | 0.37741 | 0.17251 |
| BAP7G | N6-Benzyladenine-7-glucoside | 0.01212 | 0.01287 | 0.00000 | 0.00000 |
| DHZ7G | Dihydrozeatin-7-glucoside | 0.04152 | 0.03553 | 0.02221 | 0.02593 |
| ACC | 1-Aminocyclopropanecarboxylic acid | 43.34716 | 27.10193 | 34.50784 | 91.54377 |
| GA15 | Gibberellin A15 | 0.54826 | 0.55581 | 0.54671 | 0.32454 |
| GA53 | Gibberellin A53 | 4.67516 | 3.36537 | 9.71984 | 3.76605 |
| GA19 | Gibberellin A19 | 2.24035 | 2.01864 | 4.31056 | 0.00000 |
| GA4 | Gibberellin A4 | 1.00832 | 1.72016 | 2.72518 | 0.00000 |
| H2JA | Dihydrojasmonic acid | 0.61743 | 0.57928 | 0.48004 | 1.14872 |
| OPDA | cis(+)-12-Oxophytodienoic acid | 43.54684 | 53.15532 | 52.61922 | 34.27200 |
| JA-Phe | N-[(-)-Jasmonoyl]-(l)-phenalanine | 0.35665 | 0.74121 | 1.42633 | 2.32539 |
| JA | Jasmonic acid | 1.47545 | 3.25690 | 2.88539 | 1.96964 |
| SA | Salicylic acid | 20.69561 | 16.12023 | 20.85060 | 27.73788 |
| SAG | Salicylic acid 2-O-β-glucoside | 84.16873 | 60.61789 | 72.40671 | 58.56665 |
| 5DS | 5-Deoxystrigol | 1.53379 | 1.36049 | 0.03276 | 0.00000 |

| **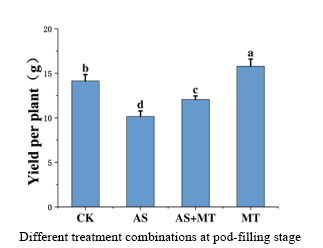** |
| --- |
| **Fig. S1** Effects of Melatonin on Soybean Yield per Plant |

| **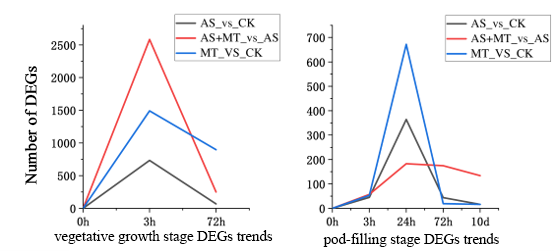** |
| --- |
| **Fig. S2** Variation trends in the number of differentially expressed genes at the vegetative growth and pod-filling stages |

| **A**  **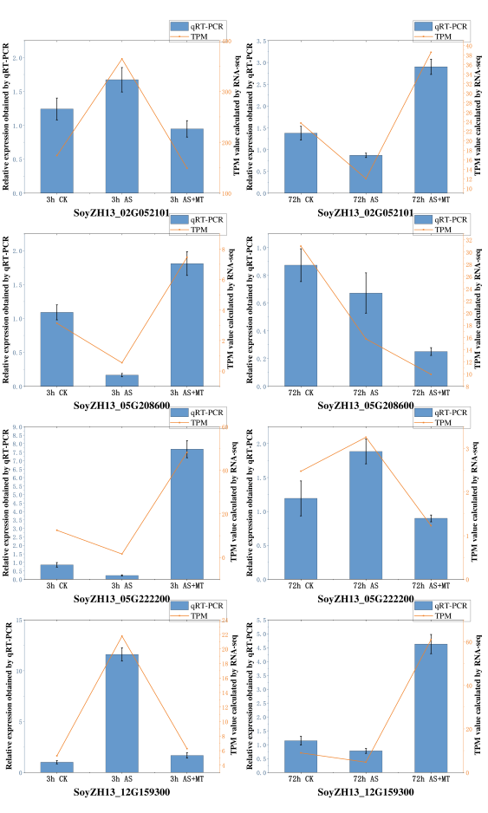** | **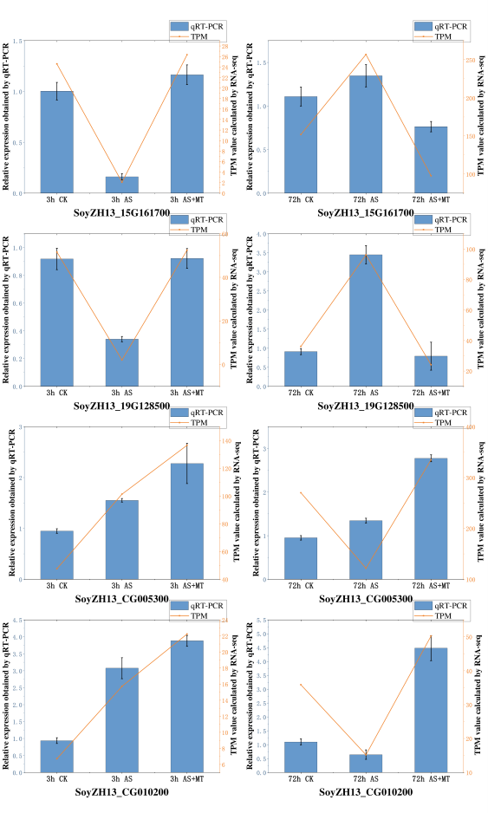** |
| --- | --- |
| **B**  **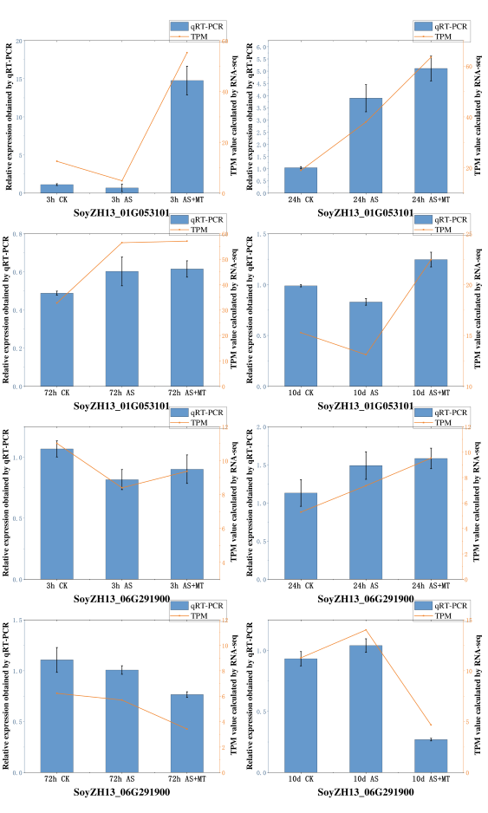** | **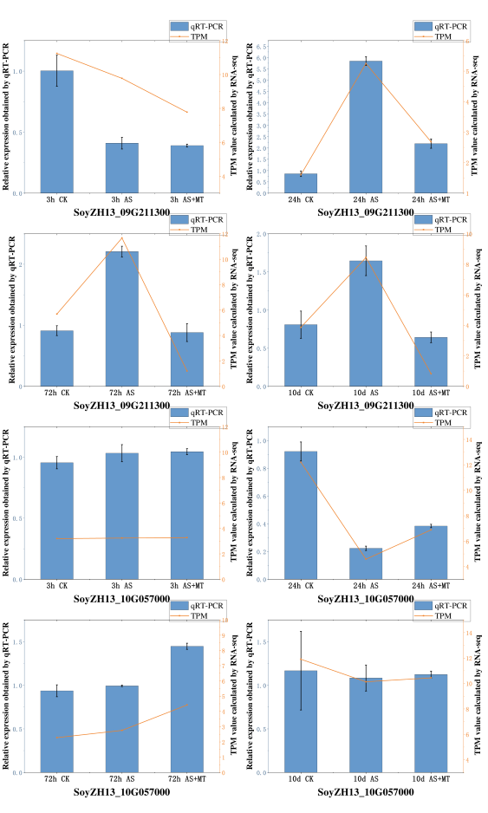** |
| **Fig. S3** qRT-PCR results. **(A)** qRT-PCR results of vegetative growth stage. (B) qRT-PCR results of pod-filling stage. Note: The left vertical axis represents the relative expression obtained by qRT- PCR analysis, which is shown by the bar graphs in the figure. Standard deviation was calculated from the results of three independent experiments. The right vertical axis is TPM value calculated by RNA-seq analysis, which is presented in the form of line graphs in the figure. Bottom is gene name and different samples at the same time point, Same below. qPCR target genes were selected based on the pathways and transcription factor family members associated with melatonin alleviation of alkali stress response in soybean. A total of 8 DEGs were selected at the vegetative growth stage (*SoyZH13_05G208600* and *SoyZH13_02G052101* are members of the MYB transcription factor family, *SoyZH13_CG005300* are genes of the photosynthesis pathway, and *SoyZH13_CG010200* are genes related to oxidative phosphorylation). A total of 8 DEGs were selected at the pod-filling stage (*SoyZH13_09G211300* is an abscisic acid signalling activation pathway related gene, *SoyZH13_11G026800* is related to flavonoid biosynthesis pathway, *SoyZH13_06G291900* is a photosynthesis pathway related gene, and *SoyZH13_10G057000* is related to alanine, aspartate and glutamate metabolism). | |
